# Multifunctional glial support by Semper cells in the Drosophila retina

**DOI:** 10.1371/journal.pgen.1006782

**Published:** 2017-05-31

**Authors:** Mark A. Charlton-Perkins, Edward D. Sendler, Elke K. Buschbeck, Tiffany A. Cook

**Affiliations:** 1 Department of Pediatrics, Division of Developmental Biology, Cincinnati Children’s Hospital Medical Center, Cincinnati, Ohio, United States of America; 2 Center of Molecular Medicine and Genetics, Wayne State University School of Medicine, Detroit, Michigan, United States of America; 3 Department of Biological Sciences, University of Cincinnati, Cincinnati, Ohio, United States of America; 4 Department of Ophthalmology, Wayne State University School of Medicine, Detroit, Michigan, United States of America; Purdue University, UNITED STATES

## Abstract

Glial cells play structural and functional roles central to the formation, activity and integrity of neurons throughout the nervous system. In the retina of vertebrates, the high energetic demand of photoreceptors is sustained in part by Müller glia, an intrinsic, atypical radial glia with features common to many glial subtypes. Accessory and support glial cells also exist in invertebrates, but which cells play this function in the insect retina is largely undefined. Using cell-restricted transcriptome analysis, here we show that the ommatidial cone cells (aka Semper cells) in the *Drosophila* compound eye are enriched for glial regulators and effectors, including signature characteristics of the vertebrate visual system. In addition, cone cell-targeted gene knockdowns demonstrate that such glia-associated factors are required to support the structural and functional integrity of neighboring photoreceptors. Specifically, we show that distinct support functions (neuronal activity, structural integrity and sustained neurotransmission) can be genetically separated in cone cells by down-regulating transcription factors associated with vertebrate gliogenesis (*pros/Prox1*, *Pax2/5/8*, and *Oli/Olig1*,*2*, respectively). Further, we find that specific factors critical for glial function in other species are also critical in cone cells to support *Drosophila* photoreceptor activity. These include ion-transport proteins (Na/K+-ATPase, Eaat1, and Kir4.1-related channels) and metabolic homeostatic factors (dLDH and Glut1). These data define genetically distinct glial signatures in cone/Semper cells that regulate their structural, functional and homeostatic interactions with photoreceptor neurons in the compound eye of *Drosophila*. In addition to providing a new high-throughput model to study neuron-glia interactions, the fly eye will further help elucidate glial conserved "support networks" between invertebrates and vertebrates.

## Author summary

Glia are the caretakers of the nervous system. Like their neighboring neurons, different glial subtypes exist that share many overlapping functions. Despite our recognition of glia as a key component of the brain, the genetic networks that mediate their neuroprotective functions remain relatively poorly understood. Here, using the genetic model *Drosophila melanogaster*, we identify a new glial cell type in one of the most active tissues in the nervous system—the retina. These cells, called ommatidial cone cells (or Semper cells), were previously recognized for their role in lens formation. Using cell-specific molecular genetic approaches, we demonstrate that cone cells (CCs) also share molecular, functional, and genetic features with both vertebrate and invertebrate glia to prevent light-induced retinal degeneration and provide structural and physiological support for photoreceptors. Further, we demonstrate that three factors associated with gliogenesis in vertebrates—*prospero/Prox1*, *Pax2*, and *Oli/Olig1*,*2*—control genetically distinct aspects of these support functions. CCs also share molecular and functional features with the three main glial types in the mammalian visual system: Müller glia, astrocytes, and oligodendrocytes. Combined, these studies provide insight into potentially deeply conserved aspects of glial functions in the visual system and introduce a high-throughput system to genetically dissect essential neuroprotective mechanisms.

## Introduction

Glia have been recognized as a major and heterogeneous non-neuronal cell type in the nervous system for more than 150 years, but their chief homeostatic and regulatory roles in nervous system development and maintenance have only recently emerged [1–4]. Despite increasing interest in the functions of glia in health and disease, the molecular networks that orchestrate gliogenesis, glial functions, and glia-neuron interactions are still enigmatic.

One of the first described glial subtypes was Müller glia. This specialized glial type is a radially-shaped macroglia that provides structural support, neuroprotection, and homeostatic recycling of energy, ions, and neurotransmitters for retinal neurons, some of the most active neurons in the body [5–7]. Müller glia are considered a specialized astrocyte, but have also been noted to share characteristics with oligodendrocytes [8,9]. Moreover, in some vertebrates (e.g. zebrafish and embryonic chick), Müller glia can serve as a source of stem cells for retinal regeneration [10], much like radial glia in other parts of the developing nervous system [11,12]. This suggests the presence of overlapping developmental and functional “networks” among different glial subtypes.

For decades, *Drosophila* has served as an effective model for uncovering conserved genetic mechanisms involved in nervous system development and physiology [4,13–15]. The fly’s visual system is among the best-characterized experimental systems for studying neuronal function and dissecting neurodevelopmental and neurodegenerative processes. In this system, a cluster of photoreceptors (PRs) in each individual eye unit (ommatidia) captures and processes light within a prominent apical compartment (rhabdomeres) that extends along the neuronal cell body and is restricted to the retina proper. Basally, the PRs project axons that exit the retina and synapse with second order neurons in the underlying optic lobe [16]. Within the optic lobe, several subretinal glial subtypes have been identified which support PR axon guidance and ensheathment, neurotransmitter recycling and neuron survival [17–21]. However, potential support roles intrinsic to the fly retina proper remain largely undefined.

**Fig 1:**
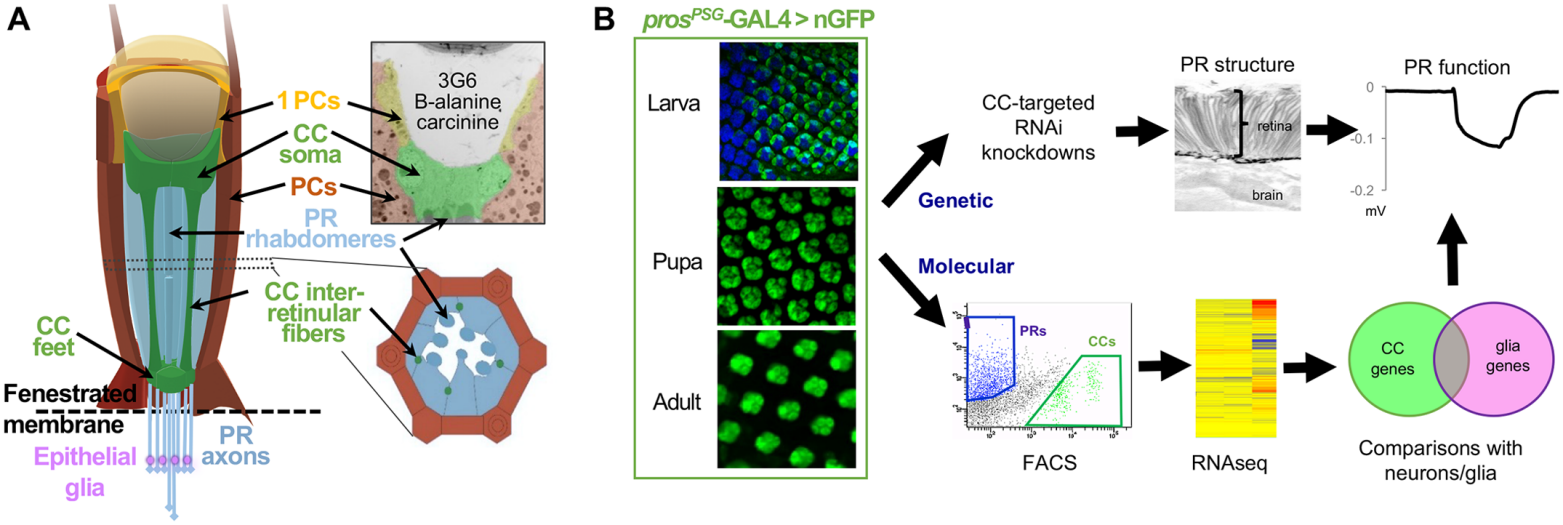
Drosophila retinal structure and expression of CC-restricted *pros*^*PSG*^-GAL4. **(A)** Diagram of an individual eye unit (ommatidium) from the *Drosophila* compound eye, highlighting CC (green)-PR (blue) interfaces throughout the retina. CCs cap the rhabdomeres distally, send interretinular fibers that contact the PR cell bodies, and form a pore at the base of the retina, around which the PR axons exit the retina. Pigment cells (PC, orange) form a fenestrated membrane which separate the retina from the brain, and through which PR axons exit the eye. Epithelial glia in the optic lobe (purple, brain) that support photoreceptor neurotransmitter recycling are also indicated. **(B)** CC expression of a *pros*^*PSG*^-GAL4 driven reporter (UAS-GFP, green) during larval, pupal, and adult stages. Elav (blue) is included in the larval stage to highlight the formation of CCs after neurogenesis in the eye imaginal disc (oriented left/anterior-to-right/posterior [youngest to oldest ommatidia]). The diagram represents the strategy of the current study, in which the *pros*^*PSG*^-GAL4 driver was used to express RNAi constructs to genetically test the role of CCs in regulating PR morphology or function and to isolate CCs for molecular (transcriptomic) analysis.

The *Drosophila* retina contains two main non-neuronal “accessory” cell types: pigment cells and cone cells (CCs) (Fig 1A) [22,23]. Pigment cells prevent light scattering between ommatidia, and have been implicated in the visual cycle, maintenance of histaminergic neurotransmitter levels, and ROS-induced lipid peroxidation [18,24,25]. These cells have also been presumed to function in ion and energy homeostasis for PRs based on electrophysiological assays in the honeybee retina [26,27].

While CCs are primarily known for their developmental role in corneal lens formation [28–31], they are also in close physical proximity to PRs, and hence are well-positioned to provide support to them. CCs radially span the retina, connecting an apical vitreous-like substance with a basal blood-retina barrier (Fig 1A) [28,30,32,33]. This configuration of corneagenous cells is highly conserved throughout arthropods, including the common house centipede in myriapods and the “living fossil” Limulus in chelicerates [34–36], raising the possibility of deeply rooted interactions between CCs and PRs. Indeed, based on such interactions, investigators speculated a glial role for CCs nearly 50 years ago [37].

Here, we present molecular and functional evidence that ommatidial CCs serve multiple glial functions in the *Drosophila* compound eye. To test this possibility, we established a CC-targeted knockdown paradigm and analyzed neighboring PRs using histological and electrophysiological methods. Genetically, we demonstrate that CCs provide structural and functional support to PRs, and that these roles are differentially contributed by transcription factors that are also involved in vertebrate gliogenesis (*Pax2*, *pros/Prox1*, and *Oli/Olig1*,*2)*. Using cell-specific transcriptomic approaches, we further document the CC-enriched expression of multiple candidate glial effector genes commonly associated with both *Drosophila* and vertebrate glia. Finally, using cell-targeted knockdowns, we demonstrate that CCs are involved in typical glial support functions, including the control of ion balance, energy resources, and sustained neurotransmission. Combined, our findings suggest that CCs serve as intrinsic retinal glia in the *Drosophila* compound eye, and establish a new, non-invasive experimental paradigm to dissect regulatory glial support modules.

## Results

### Cone cells control retinal structure via *Pax2*

Developing and adult CCs specifically express *prospero* (*pros*) and *dPax2* (a.k.a *sv*) [31,38,39] (S1A Fig), two transcription factors widely associated with glial development and/or function [40–54]. Previous studies on the role of *pros* and *dPax2* in CCs showed that these factors function cooperatively to distinguish the non-neuronal CC fate from the fate of the last neuronal cell type (the R7 photoreceptor) via feedback control of Ras and Notch signaling [31], a feature also common for neuron-glia fate decisions [55–60]. Importantly, individual *pros* and *dPax2* mutants minimally affect CC specification [31,39], allowing us to test the hypothesis that CCs serve glial-like support role in the fly retina through these factors.

**Fig 2:**
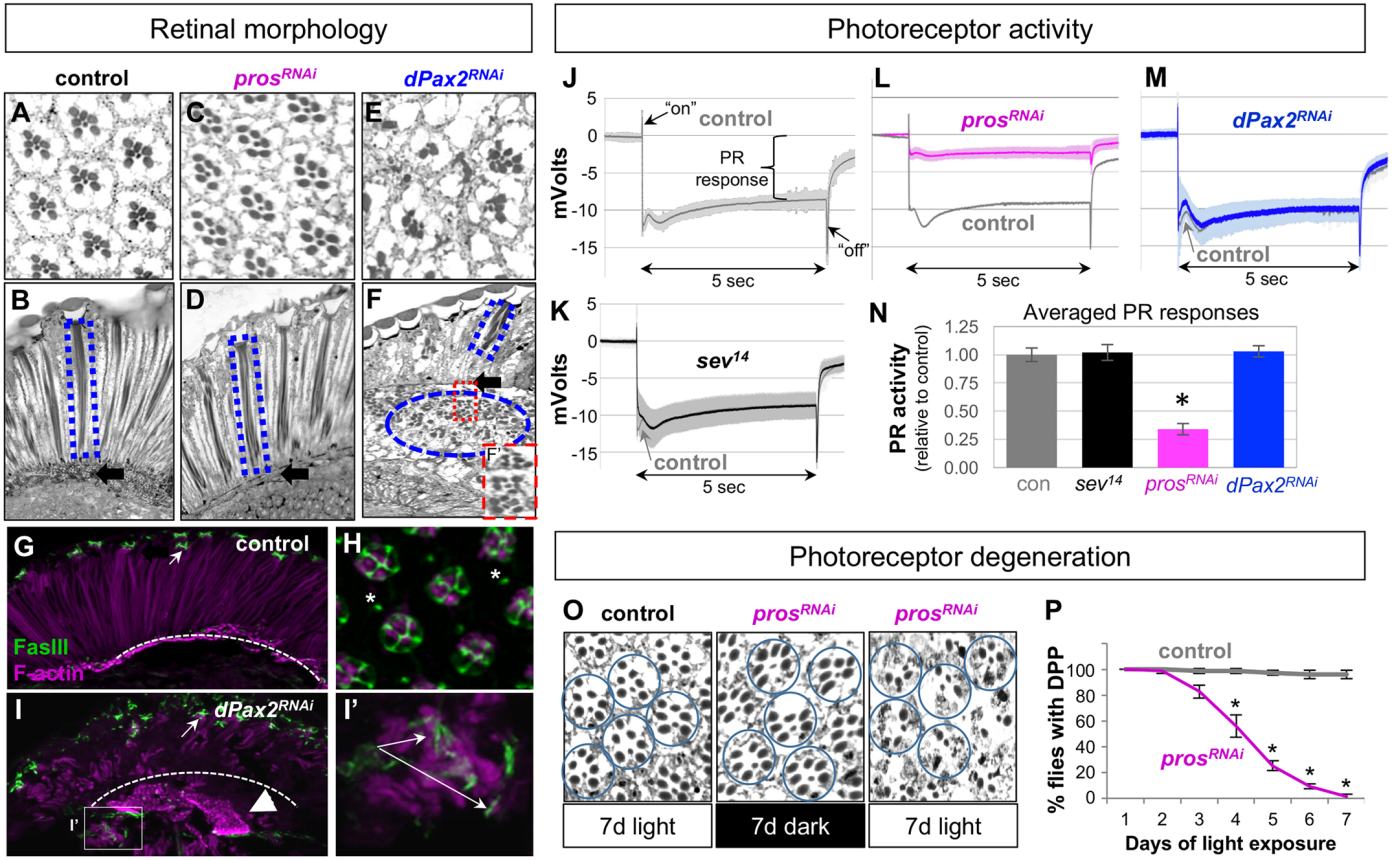
Histological and electrophysiological evidence for CCs providing structural and functional support for *Drosophila* photoreceptors. **(A-F)** Toluidine blue-stained coronal (A,C,E) and sagittal (B,D,F) thin sections from adult control (*pros*^*PSG*^-GAL4>UAS-*nGFP*, A, B), *pros*^*RNAi*^ (*pros*^*PSG*^-GAL4>UAS-*pros*^*RNAi*^, C, D), and *Pax2*^*RNAi*^ (*pros*^*PSG*^-GAL4>UAS-*dPax2*^*RNAi*^, E,F) eyes. In B,D,and F, the blue dashed lines highlight rhabdomeres and filled arrow indicates the fenestrated membrane. F’ magnifies a subretinal region in *Pax2*^*RNAi*^ heads, highlighting relatively intact PR clusters (**G-I)** CCs (marked with Fas3, green, arrows) remain associated with PRs (marked with phalloidin, magenta) in both control and *dPax2*^*RNAi*^ flies, even though many PRs have fallen into the brain in *dPax2*^*RNAi*^ flies (I, I’). The interommatidial bristle (*) also expresses Fas3 (H) in adult eyes. **(J-M)** Averaged ERGs from dark-adapted control (*pros*^*PSG*^-GAL4; UAS-*nGFP*), *pros*^*RNAi*^ (*pros*^*PSG*^-GAL4; UAS-*pros*^*RNAi*^), and *Pax2*^*RNAi*^ (*pros*^*PSG*^-GAL4; UAS-d*Pax2*^*RNA*i^) excited with 5x5sec pulses of 490 nm (blue-green) light (n = 5 animals). Standard deviation is shown by the shaded area, and day-matched control recordings are included in grey for each experimental condition. **(N)** Averaged PR responses from J-M, normalized to day-matched controls. *C*control, *sev*^*14*^, and *dPax2*^*RNAi*^ flies are indistinguishable, while *pros*^*RNAi*^ flies have significantly reduced PR responses (*p<0.001). **(O)** Control or *pros*^*RNAi*^ flies raised for 7 days in dark or light and PR structure were analyzed by toluidine blue stained semi-thin plastic sections, showing a light-dependent degeneration only in *pros*^*RNAi*^ flies raised under continuous light. **(P)** DPP presence was quantified daily for 7 days from control or *pros*^*RNAi*^ flies raised in continuous light (* p<0.001 by day 3, n = 3 replicates of 25 flies each). Error bars = S.D.

For these studies, we used the GAL4-UAS system to drive *pros* and *dPax2*-directed RNAi constructs in CCs and tested for the phenotypic consequences on neighboring neuron morphology and activity as 2 measures of common glial support functions. GAL4 expression was driven using a 275 bp *pros* enhancer that is expressed in CCs and R7s from early specification through adulthood, with little to no expression in the underlying optic lobe (Fig 1B, S1B and S1C Fig). Importantly, we confirmed that animals lacking R7s (*sev*^*14*^ mutants) do not exhibit the morphological or electrophysiological phenotypes reported here [31,61–63] (Fig 2K and 2N), allowing us to conclude that the ERG and morphology phenotypes described below are dependent on CC and not R7 function.

We first histologically analyzed retinal integrity in adult eyes knocked down for *pros* and *dPax2* using previously verified RNAi constructs [31]. In wild type and control (*pros*^*PSG*^*-GAL4>nGFP*) animals (Fig 2A), the regularly spaced ommatidia centrally house a trapezoidal array of actin-rich rhabdomeres from the main PR neurons (R1-R6).The rhabdomeres and PR cell bodies span the depth of the retina, are caged by CCs, and are physically segregated from the brain and PR axons through a fenestrated membrane formed by the interommatidial pigment cells (Figs 1A, 2B and 2G, S2A Fig) [32]. Retinas from *pros*^*RNAi*^ CC knockdowns appeared morphologically similar to controls (Fig 2C and 2D; S2B Fig), while *dPax2*^*RNAi*^ knockdowns showed severe defects in retinal and PR rhabdomere morphology (Fig 2E, 2F and 2I; S2C Fig). These results are consistent with previous results using eye-specific mutants for these factors (*pros*^*17*^ and *sv*^*spapol*^) [31,38,39,64,65]. Also similar to lens defects in *dPax2*^*spapol*^ mutants [31,39,65], *dPax2*^*RNAi*^ CC knockdowns showed a variable severity in retinal defects (Fig 2I, S2C Fig). This ranged from moderately affected regions with short, misshapen rhabdomeres that failed to extend through the retina (Fig 2F and 2I; S2C Fig) to more severe regions in which clusters of rhabdomeres were mislocalized beneath the fenestrated membrane, hence absent from the retina (Fig 2E, 2F, 2F’ and 2I; S2C Fig) [65]. Interestingly, rhabdomere clusters observed in the brain maintain a relatively normal ommatidial arrangement (Fig 2F’), suggesting that fully formed eye units had lost contact with the retina.

To test if *dPax2*-negative CCs remained in contact with PRs, we performed immunostaining for Fasciclin 3 (Fas3), a transmembrane protein we identified in a larger screen for CC markers. In control eyes, Fas3 is exclusively localized to CC-CC interfaces as well as the interommatidial mechanosensory bristle (Fig 2G and 2H). In *dPax2*-negative CCs, Fas3 immunostaining remained closely associated with actin-rich PR rhabdomeres at the top of the retina as well as with the mislocalized PRs beneath the retina (Fig 2I and 2I’). Together, these findings suggest that *dPax2* is required in CCs for proper elongation of PR rhabdomeres and overall retinal architecture, but not for the intimate association of CCs and PRs.

### Cone cells regulate photoreceptor function via *prospero*

We next assayed the neuronal activity of PRs in *pros*^*RNAi*^ and *dPax2*^*RNAi*^ CC knockdown flies using electroretinogram (ERG) recordings (Fig 2J–2N). Like wild-type animals [66,67], dark-adapted control flies (*pros*^*PSG*^>*GFP*) (Fig 2J) and *sev*^*14*^ flies (Fig 2K) exhibited a strong PR-dependent depolarization (~10 mV) in response to light, as well as second-order neuron responses in the optic lobe, detectable as “on” and “off” peaks (transients) before and after PR depolarization. Similar normal ERG traces were recorded from *dPax2*^*RNAi*^ CC knockdowns (Fig 2M), suggesting that despite the disruption in retinal architecture, the PRs in *dPax2*^*RNAi*^ knockdown flies remain functional. In marked contrast, *pros*^*RNAi*^ CC knockdowns showed a significant reduction in overall PR depolarization in dark-adapted flies (Fig 2L and 2N; S3A Fig) and almost no PR response in light-adapted flies (S2D Fig), suggesting substantial visual loss. Importantly, eyes fully mutant for *pros* (*Minute* clonal analysis with *pros*^*17*^) showed a similar reduction in PR activity as *pros*^*RNAi*^ CC knockdown flies (S2D Fig). No changes in the “on” transient peak was observed in *pros*^*RNAi*^ CC knockdowns or *pros*^*17*^ mutant retinas (S3B Fig) when normalized to overall PR activity, suggesting that second-order neuronal activation is intact in these animals. Combined, these electrophysiological analyses reveal that *pros*, and not *dPax2*, is necessary in CCs to sustain proper PR function. Further, these data indicate that CC-dependent support of PR structure and function are genetically separable by *pros* and *dPax2*.

### *Prospero* is required in cone cells to prevent light-induced neurodegeneration

Like vertebrate PRs, *Drosophila* PRs are susceptible to excitotoxic injury and degeneration under sustained bright light conditions [68–72]. While the abnormal retinal morphology in *dPax2*^*RNAi*^ flies prevented reliable evaluation of light-induced degeneration, the healthy appearance of *pros*^*RNAi*^ CC knockdowns under normal lab-rearing conditions allowed us to test for a possible role of CCs (and *pros)* in this process. Histological examination of control animals (*pros*^*PSG*^*>GFP*) raised 7 days in continuous darkness or sub-degenerative light conditions [72] displayed similarly intact rhabdomere morphology (Fig 2O, S2E and S2H Fig), as did dark-raised *pros*^*RNA*i^ flies (Fig 2O, S2F and S2I Fig). In contrast, *pros*^*RNAi*^ flies raised under continuous light showed signs of rhabdomere degeneration (Fig 2O, S2G and S2J Fig).

To quantify this *pros-* and light-dependent retinal degeneration, we made use of the deep pseudopupil (DPP) of the *Drosophila* compound eye. Visualization of the DPP allows for non-invasive detection of intact rhabdomere integrity in living flies, and its presence declines in flies undergoing retinal degeneration [71,73,74]. Consistent with the above histological analysis, DPP monitoring in animal populations raised for 7 days in continuous light showed intact DPPs in >95% control flies throughout the course of the experiment, whereas DPP loss was observed in >50% of *pros*^*RNA*i^ flies by day 3 and in all *pros*^*RNA*i^ flies by day 7 (Fig 2P). Control and *pros*^*RNA*i^ flies raised in total darkness also preserved DPPs throughout the experiment. Combined, these findings indicated that *pros* in CCs is essential for preventing light-dependent degeneration of PRs.

### Drosophila cone cells express and require key gliogenic genes for photoreceptor support

To molecularly define CCs, we turned to a cell type-specific transcriptomic approach. For these experiments, we isolated CCs by fluorescence-activated cell sorting (FACS) from retinal tissue dissected at three developmental stages: specification (larval CCs), maturation/differentiation (pupal CCs), and terminal differentiation/maturity (adult CCs) (see Methods). Adult PRs were isolated for comparisons (S4A Fig). RNA isolated from these sorted cell populations was sequenced using Illumina HiSeq2500, and transcript quantification using TMM normalization was applied to remove RNA compositional biases between samples and improve the compatibility of cross-sample analysis (S1 Table). Validation experiments confirmed that multiple housekeeping genes were expressed equivalently across all transcriptomes, and that known PR differentiation and effector genes (*e*.*g*. *oc/Otd*, *ninaE/Rh1*, *Arr1 and chaoptin*) were specifically enriched in PRs and not CCs (S4B Fig). In addition, proteins previously shown to be expressed in mature CCs (e.g. *cut* (*ct*), *eyes absent (eya)*, *and Drosocrystallin/Crys*) [29,75,76] were among the top 50 cell-specific genes expressed in our adult CC transcriptome (S1 Table).

**Fig 3:**
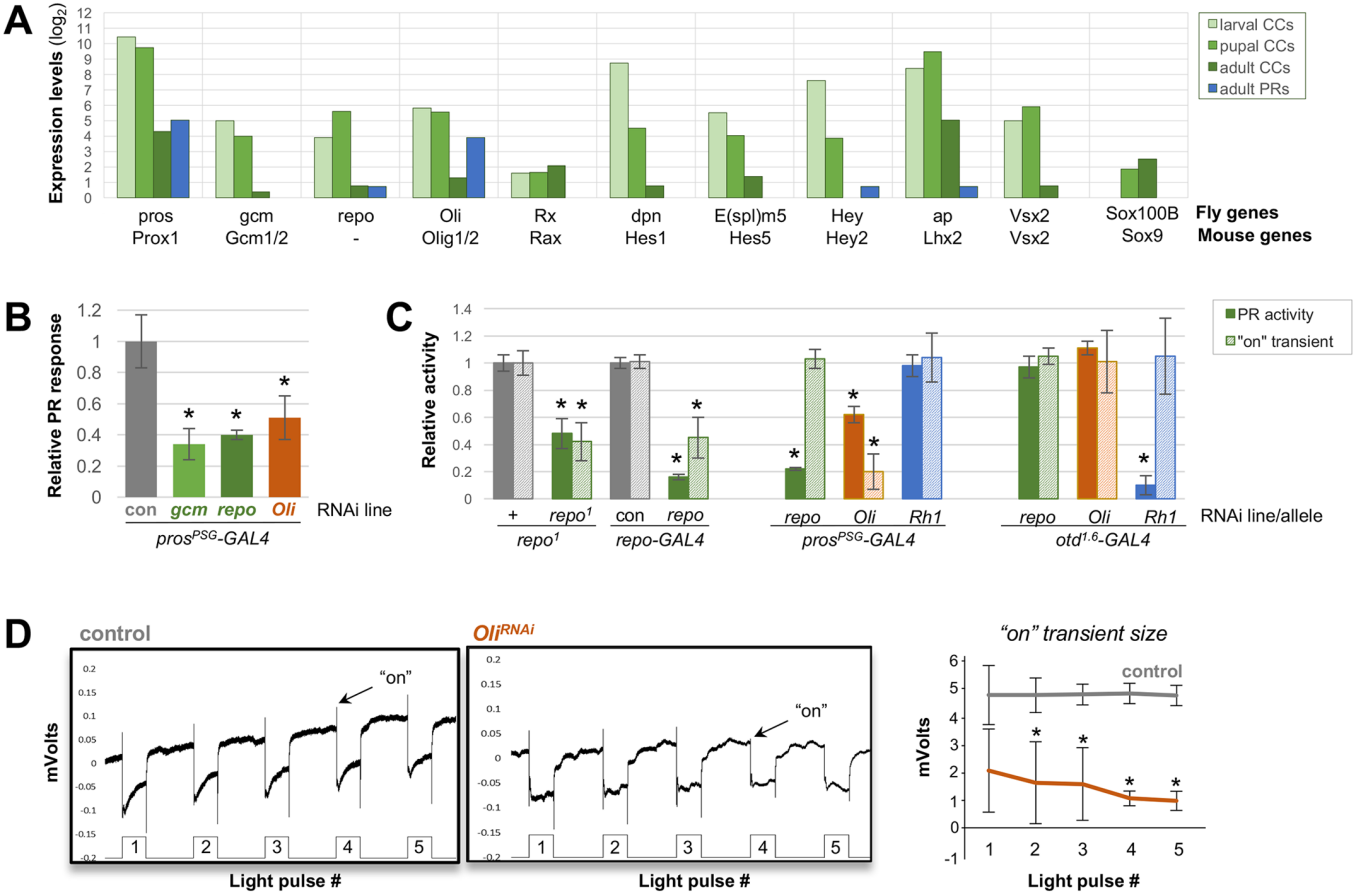
Gliogenic genes are expressed and required in *Drosophila* cone cells for photoreceptor activity. **(A)** TMM-normalized log_2_ expression levels of transcription factors frequently associated with fly and vertebrate glial specification, plotted from larval, pupal and adult CCs (green) and adult PRs (blue). **(B)** Relative PR activity (normalized to day-matched controls, see Methods and S4A and S4B Fig) from ERG recordings of *pros*^*PSG*^-GAL4 > nuclear GFP (con), and knockdowns using lines for *pros*^*PSG*^*-GAL4* driven *gcm*^*RNAi-1*^, *repo*^*RNAi-1*^, and *Oli*^*RNAi-1*^ lines indicate that all three factors are required in CCs for full PR activity. **(C)** Relative PR activity (solid bars) and relative “on” transient measurements (striped bars) from heterozygous or homozygous *repo*^*1*^ animals, or *pros*^*PSG*^- (CC-), *repo*- (glia-), and *otd*^*1*.*6*^- (PR-) GAL4 lines driving *gcm*^*RNAi-1*^, *repo*^*RNAi-1*^, *Oli*^*RNAi-1*^, and the *Rh1/ninaE*^*RNAi-1*^ flies (n = 5 flies, 5x5sec light flashes each). “on” transients were calculated as the ratio of the “on” transient strength to the maximal PR response (see Methods and S4C Fig). *N*.*B*. We did not detect the early reversed polarity phenotype initially reported in very young *repo*^*1*^ flies (<1 d old [78]), but instead observed the phenotypes observed with older *repo*^*1*^ flies [97]. **(D)** Relative “on” transient size from each of 5 light pulses from control and *pros*>*Oli*^*RNAi-1*^ flies tested for significance by 1-way ANOVA. Error bars = SEM, * p<0.001.

Given the critical roles of *glial cells missing/glide* (*gcm)* and *reversed polarity* (*repo*) in the determination of most *Drosophila* glial cell fate decisions [77–81], we first investigated their presence in our CC transcriptome data. Expression of both *gcm* and *repo* was appreciably higher in developing CCs compared with adult CCs and PRs (S1 Table, Fig 3A). Peak *pros* and *gcm* levels preceded that of *repo* (Fig 3A), consistent with previous findings that *pros* activates *gcm*, which in turn activates *repo* in subpopulations of *Drosophila* glia [44,49,58,82–85]. The transient expression of *gcm* and *repo* during CC development was further confirmed with immunostaining for a *gcm* reporter construct and Repo protein (S4C–S4F Fig). Thus, both gliogenic genes associated with the *Drosophila* nervous system are transiently expressed in developing CCs.

We next examined our larval, pupal, and adult CC transcriptomic data for the presence of orthologous factors that, like *pros/Prox1* and *Pax2*, are commonly associated with gliogenesis in the vertebrate nervous system. This analysis showed transient expression of the transcription factors *Olig1*,*2/Oli*, *Rx/Rax*, *Hes1/dpn*, *Hes5/E(spl)*, *Hey2/Hey*, *Lhx2/ap*, and *Vsx2/Vsx2* in larval and pupal, but not adult, CCs (Fig 3A). *Oli* expression was also specifically detected in PRs, consistent with previous studies reporting fly and vertebrate Oli gene expression in both early neural progenitors and specified neurons [86–89]. Further, we detected *Sox100B* expression in pupal and adult (but not larval) CCs (Fig 3A, S1 Table), similar to the later onset of expression found for its ortholog *Sox9* in vertebrate retinal glia [90,91]. These findings hence revealed that the developing CCs of the *Drosophila* compound eye express multiple, deeply conserved gliogenic factors.

To functionally test whether candidate gliogenic genes besides *pros* and *Pax2* are required in CCs for PR support, we focused on *gcm* and *repo* as *bona fide Drosophila* glial factors, and *Oli*, a known gliogenic gene in vertebrates and *C*. *elegans* [89,92–94], but whose function in *Drosophila* has thus far only been analyzed in motor neuron development [86]. Applying the same targeted RNAi /ERG strategy as above, we tested two independent RNAi lines for each candidate gene, each pair yielding comparable results (S3A Fig). Cone cell specificity was tested using either pan-photoreceptor (*otd*^*1*.*6*^-GAL4) [95] or pan-glial (*repo*-GAL4) [96] drivers. All *gcm*, *repo*, and *Oli* RNAi knockdowns developed eyes with a normal appearance of external lens facets and DPPs, indicating that similar to *pros*, depletion of these factors in the eye does not demonstrably affect cell type specification or ocular morphogenesis.

ERG recordings from either *gcm* or *repo* CC knockdown animals (*pros>gcm*^*RNAi*^, *pros>repo*^*RNAi*^) revealed a similar phenotype as *pros>pros*^*RNAi*^ flies: a significant reduction in PR activity (~60% below control values) (Fig 3B and 3C; S3A and S5 Figs) and no detectable changes in normalized transient sizes (S3C and S3D Fig). These results are consistent with *gcm* and *repo* lying downstream of *pros* in subsets of other developing glia [40,42,44,49]. A similar *repo*-dependent reduction in PR activity was previously reported for viable adult *repo*^*1*^ hypomorphs [78,97]. Notably, however, unlike our CC knockdowns of *repo*, *repo*^*1*^ mutants also exhibit reduced “on” transients [78,97] (Fig 3C), suggesting defects in optic lobe glia. To validate the effectiveness of our *repo*^*RNAi*^ approach, we 1) confirmed that Repo protein levels in *pros*>*repo*^*RNAi*^ knockdown ommatidia were specifically reduced in CCs (S4F Fig), and 2) knocked down *repo* in all glia using *repo*-GAL4. In agreement with studies using null *repo* alleles, *repo*>*repo*^*RNAi*^ animals raised at our normal experimental conditions (25°C) showed early lethality, further confirming efficient *repo* knockdown [77,79]. In addition, knocking down *repo* later during eye development, making use of the temperature-sensitivity of GAL4 (see Methods), led to both *repo*^*1*^-associated phenotypes [97]: reduced PR activity and “on” transients (Fig 3C; S3B–S3D Fig). Other cell specificity control experiments showed that PR-specific *repo*^*RNAi*^ expression had no effect on ERG activity, while PR knockdown of *ninaE*/*Rh1* (the primary opsin contributing to ERG activity) reduced PR activity by >90% and CC knockdowns of *Rh1* showed no changes in PR activity (Fig 3C; S3A Fig). Combined, these data indicate that *gcm* and *repo* are required during CC development to promote support function(s) in the adult retina whereas *repo*-positive cells outside of the retina support “on” transient activity.

Like *pros*, *gcm*, and *repo* knockdowns, ERG recordings of *Oli* CC knockdowns also showed a reduction in overall PR depolarization (~40% of control levels) (Fig 3B). In marked contrast to these other knockdowns however, *Oli*^*RNAi*^ CC knockdown flies also showed a reduction in the average “on” transient responses (Fig 3C and 3D; S3B Fig). Further analysis of this outcome revealed a light-pulse dependent decay of the transient size (Fig 3D, S3D Fig), a phenotype not observed with any of our other CC knockdowns. Selective knockdown of *Oli* in PRs on the other hand, resulted in ERG traces comparable to control animals (Fig 3C; S5A Fig). These combined results suggest that the transient expression of *Oli* specifically in *Drosophila* CCs regulates developmental networks required for neuronal activity and sustained neurotransmission in neighboring PRs.

### Drosophila cone cells are enriched in fly glia-associated genes

**Fig 4:**
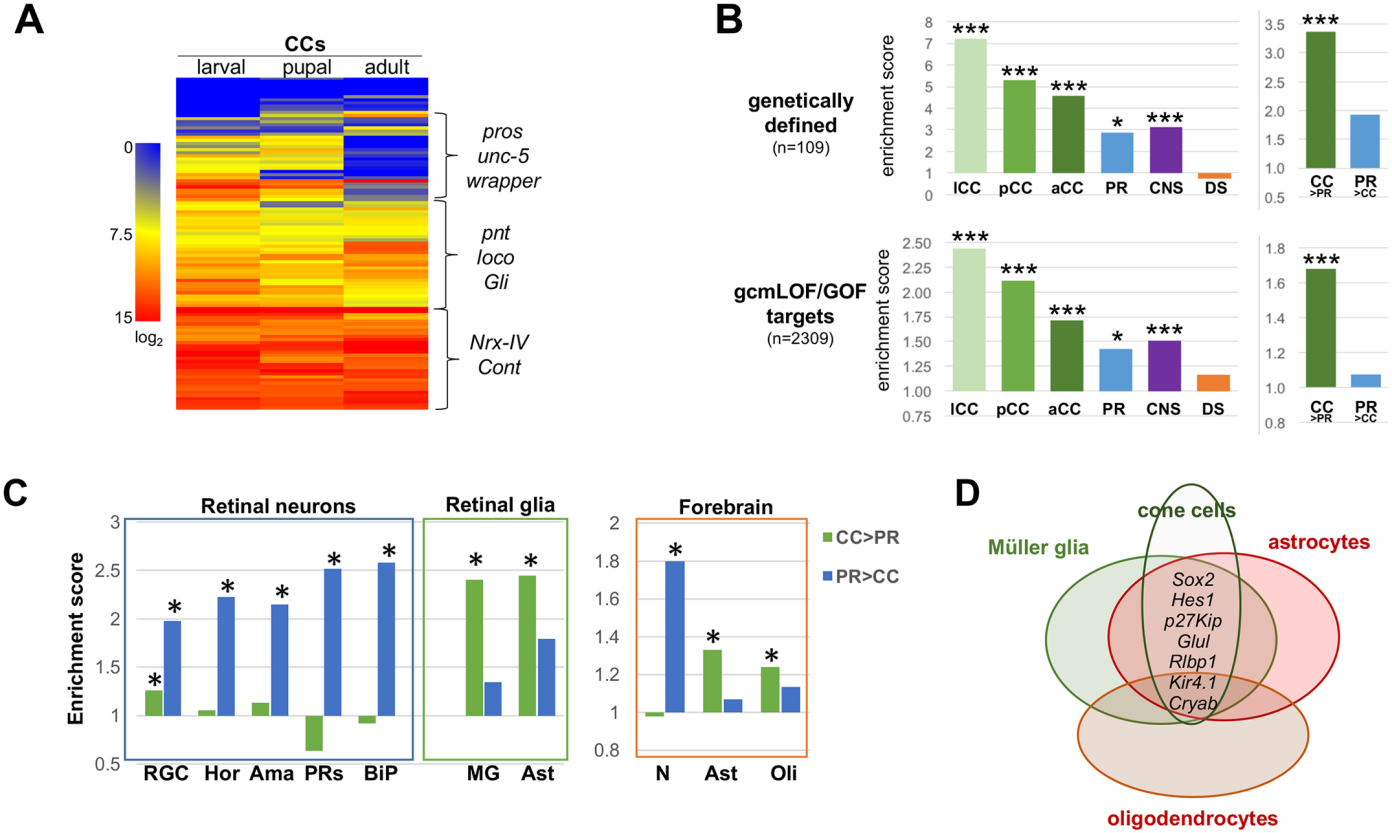
Transcriptome comparisons with Drosophila cone cells/photoreceptors and glial/neuronal cell populations. **(A)** Heatmap of TMM-normalized log_2_ expression levels from larval, pupal and adult CCs for 109 genetically-identified factors involved in fly glial differentiation (http://www.sdbonline.org/sites/fly/aimorph/glia.htm) [98]. Examples of genes that cluster based on different expression dynamics or levels are indicated by brackets. **(B)** Relative overlap of genes present in different cell and tissue types [1000 genes with highest relative expression] in larval, pupal, and adult CCs (green), PRs (blue), mature nervous system (purple), and mature digestive system (orange)] with 109 “glial” genes [98] or 2309 *gcm* downstream factors [those deregulated in both loss- and gain-of-function *gcm* experiments [84]]. Separate analysis (right panels) using the 1000 most CC- vs. PR-enriched sets (CC>PR, PR>CC) shows specific enrichment for glial genes in CCs but not PRs. Enrichment = actual number of overlapping genes/expected number of overlapping genes; *p<0.02, ** p<0.002. **(C)** Relative enrichment values (as in **B**) for Drosophila CC vs PR-enriched genes analyzed for overlap with murine retinal and forebrain neuronal and glial cell types. **(D)** Venn diagram representing commonly associated glial genes between cone cells, Müller glia, astrocytes, and oligodendrocytes.

To further probe the potential glial properties of CCs, we first identified the top 1000 most enriched genes expressed in developing, differentiating, and mature CC gene sets (larva, pupal, and adult CCs, S2 Table). We then compared these CC-enriched gene sets with 109 genes previously identified in a genetic screen for roles in *Drosophila* glial differentiation (http://www.sdbonline.org/sites/fly/aimorph/glia.htm) [98] (S3 Table). Such analysis revealed that 49% (53/109) of these genetically-defined glial genes are prominent in at least one stage of CC development. These included factors known to function downstream of *pros*, *gcm* and *repo* (e.g. *pnt*, *loco*, *and unc-5*) and cell adhesion molecules commonly associated with glia (e.g. *wrapper*, *gliotactin* (*Gli*), *neurexin IV* (*NrxIV)*, and *Contactin (Cont))* (Fig 4A) [40,77,82,99–105].

To assess the cellular specificity of glial gene expression in CCs, we constructed corresponding enriched gene groups derived from adult PRs (from this study) and publicly-available central nervous system (CNS) and digestive system (DS) RNA-seq data sets (see Methods). When compared to the 109 glial genes analyzed above, larval CCs showed the highest enrichment, followed by pupal CCs, adult CCs and the CNS (Fig 4B). Of the neural cell populations analyzed, PRs showed the least significant enrichment, while consistent with its non-neural origin, the DS showed no significant enrichment. Similar enrichment patterns were observed when these samples were compared with 2309 *Drosophila* genes regulated by *gcm* in both loss- and gain-of-function paradigms [84] (Fig 4B, S3 Table). Finally, comparison of the top 1000 genes most differentially expressed between adult CCs and PRs with both *Drosophila* glial gene sets showed a significant enrichment of CC-, but not PR-specific, genes (Fig 4B). Combined, these molecular analyses indicate that fly CCs are specifically enriched for a broad panel of fly glia-associated gene products.

### Adult fly cone cells share molecular signatures with vertebrate glia

Having established the expression of many developmental factors associated with vertebrate gliogenesis in developing *Drosophila* CCs, we next tested for overlap of our adult CC- and PR-specific gene sets with published glial- or neuronal-restricted effector genes from postnatal mouse retina and forebrain [106–108]. This analysis revealed that mouse orthologs of genes highly expressed in fly PRs were significantly enriched in all mouse neuronal cell populations analyzed, while those genes expressed in fly CCs showed the highest enrichment for murine astrocytes and Müller glia (Fig 4C). Lower but significant enrichment was also observed between CCs and oligodendrocytes as well as retinal ganglion cells (Fig 4C). Consistent with an enrichment of Müller glia and astrocyte genes in CCs, several common astroglial markers showed overlap among these three cell populations, including *Sox2*, *Hes1*, *Cdkn1(p27Kip)*, *Glul(GS)*, and *Rlbp1(CRALBP1)* (Fig 4D, S5 Table). In addition, the Kir4.1 inward rectifying potassium channel (*Kcjn10*) and the chaperone *Cryab* were shared among the CC-, Müller glia-, astrocyte- and oligodendrocyte-enriched gene sets (Fig 4D, S5 Table). These data suggest that fly retinal CCs share molecular signatures with Müller glia, astrocytes, and to some degree, oligodendrocytes.

### Fly cone cells provide ion and energy support for PRs via established glial effector genes

**Fig 5:**
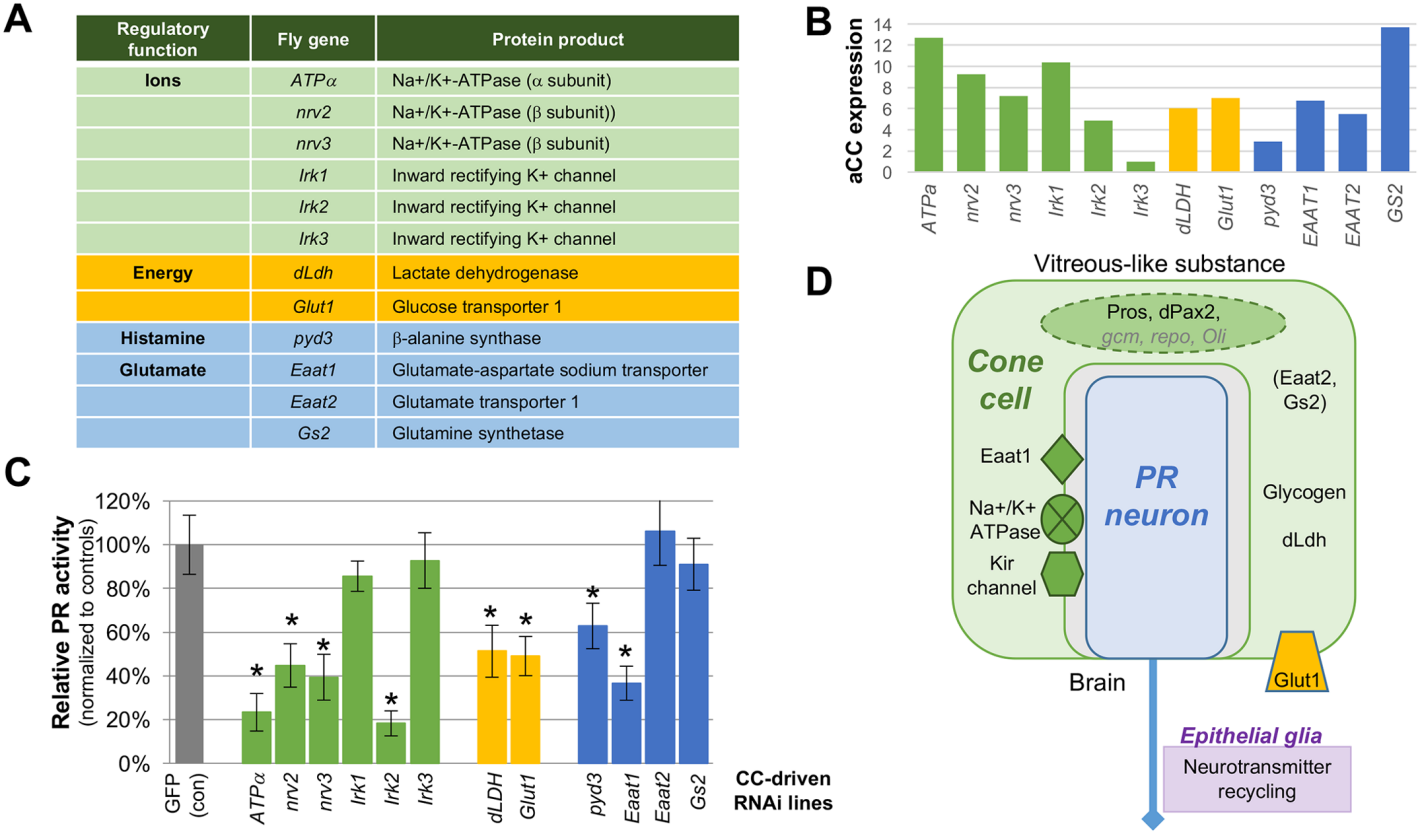
Functional dependence of cone cell-expressed glial effectors on photoreceptor physiology. **(A)** Table of 12 conserved glial genes tested for CC-dependent PR activity support functions. **(B)** TMM-normalized RNA expression levels of candidate genes from (C) in adult cone cells. **(C)** Relative PR activity (normalized to day-matched controls, +/- SEM) for CC knockdowns with *pros*^*PSG*^-GAL4 (*p<0.001). Eight show significant reductions in PR activity, including those that are typically associated ion maintenance (*Atpα*^*RNAI-1*^, *nrv2*^*RNAI-1*^, *nrv3*^*RNAi-1*^ and *Irk2*^*RNAi-1*^), metabolism (*dLDH*^*RNAi-1*^ and *Glut1*^*RNAi-1*^) and histamine and glutamate recycling (*pyd3*^*RNAi-1*^ and *EAAT1*^*RNAi-1*^). Four (*Irk1*^*RNAi-1*^, *Irk3*^*RNAi-1*^, *EAAT2*^*RNAi-1*^ and *GS2*^*RNAi-1*^) were statistically similar to controls. **(D)** Schematic of the glial factors expressed in and required by CCs for supporting retinal integrity and function. The transient expression of *gcm*, *repo*, and *Oli* are reflected by lighter font. Parentheses indicate glial factors with abundant mRNA expression in CCs, but whose function was not defined here. The role of epithelial glia in neurotransmitter recycling, a synaptic event that occurs in the brain and which we found to be genetically separable from CCs, is also noted.

To functionally test for potential glial homeostatic functions in CCs, we focused on a subset of 11 candidate effectors: α- and β-subunits of the Na/K-ATPase (a.k.a. the Na+ pump), K-inward rectifying channels (*Kir2*.*1/4*.*1* homologs), lactate dehydrogenase (*dLdh/Impl3*), glucose transporter 1 (*Glut-1*), the excitatory amino acid transporters *Eaat1/Glast and Eaat2/Glt-1*, and the glutamate-ammonia ligase glutamine synthetase (*GS2*) (Fig 5A). Notably, these genes promote three important support functions that are conserved in the insect and vertebrate retina—ion balance, energy homeostasis, and glutamate recycling [109]. In addition, these genes are commonly used to identify vertebrate glial types, including Müller glia, astrocytes, and oligodendrocytes. Based on our transcriptome analysis, orthologs for each of these factors were expressed at relatively high levels in adult CCs (Fig 5B). Also, because fly PRs are histaminergic and the CC-generated pseudocone serves as a reservoir for the histamine-associated metabolites β-alanine and carcinine, we analyzed β-alanine synthase/β-ureidopropionase (*Drosophila pyd3*/vertebrate *UPB/BUP1*), a deeply conserved factor necessary for histamine recycling, pyrimidine and vitamin B biogenesis, energy production, and antioxidant production [110–112]. Again, 2 independent RNAi lines were tested for each gene, both showing comparable effects on PR activity (S3C Fig). Cell-specific knockdowns for a subset of factors was confirmed by immunostaining (S6 Fig), and adult eyes from each CC knockdowns lacked detectable external defects in lens or PR morphogenesis.

CC knockdowns for the subunits for the Na+ pump (*ATP*α, *nrv2*, *nrv3)*, as well as the Kir channel *Irk2* resulted in significantly reduced PR responses relative to controls (Fig 5C). In contrast, knockdown of *Irk1* or *Irk3* showed ERG recordings comparable to controls (Fig 5C). CC knockdowns of energy-promoting factors *dLdh* and *Glut1* each led to a ~50% reduction in overall PR responses (Fig 5C; S3C Fig). And finally, CC knockdown of *Eaat1* or *pyd3* led to a significant reduction in PR activity, while *Eaat2* or *Gs2* CC knockdowns showed control levels of PR activity (Fig 5C; S3C Fig). We did not detect significant changes in averaged “on” transient size for any of the effector genes tested (S3B and S3D Fig). Combined, these data demonstrate that CCs require multiple but distinct glial effectors to specifically promote PR neuronal activity.

## Discussion

The multifunctional roles played by glia to support neuron development, activity, and integrity are best characterized in vertebrates, where subtypes as diverse as astrocytes, oligodendrocytes, radial glia, tanycytes, and microglia perform specific subsets of these specialized developmental and homeostatic functions [8,9,11,12]. In the invertebrate nervous system, fewer but similar accessory and supportive glial cell types have been recognized [15,113–115]. Yet the degree to which fly glial types relate to vertebrate glial lineages requires further study [4,116]. Drawing on molecular, genetic, and physiological evidence, our studies document a novel class of invertebrate glia, the CCs of the *Drosophila* compound eye. The further discovery, using genome-wide transcriptome analysis, that CCs share molecular and functional fingerprints with vertebrate glial types like Müller glia and astrocytes opens the door to non-invasive high-precision analyses of glia-neuron interactions in *Drosophila*. Moreover, our findings offer new insight on the evolution of deeply conserved gene networks (“character networks”) [117] that are important for both vertebrate and invertebrate support cells.

### “Glial character networks”

Recent advances in evolutionary biology have come to recognize deeply conserved gene networks [117] (also termed “kernels” [118] or “character identity networks” [119]) as a valuable means to detect and explore structural and functional homologies among tissues and cell types across animal phyla [120–122]. In the current study, we have identified several support roles that cone cells provide to their neighboring retinal neurons. Such roles include ionic and metabolic homeostasis, identified through functional tests of classic glial effectors such as the Na+/K+ ATPase, Kir channels, Eaat1, Ldh, and Glut-1. Our study complements seminal work in the honeybee compound eye, which based on electrophysiological and histological findings, already predict the use of such factors in undefined “retinal glia” [26,109]. More recent genetic studies in *Drosophila* demonstrated that interommatidial pigment cells mediate other support roles, including neurotransmitter storage, visual pigment recycling, and lipid peroxidation [18,24]. Combined with the present findings, these data suggest that both accessory cell types in the insect compound eye—CCs and interommatidial pigment cells—serve glial functions that resemble the overlapping support roles of Müller glia and the retinal pigmented epithelium in the vertebrate retina [7,123].

Complementing the homeostatic functions performed by CCs, our studies further reveal at least three genetically distinct transcriptional inputs that are pertinent to CC-dependent retinal support. Knockdowns for *pros*, *gcm*, and *repo*, for instance, show a requirement for this known gliogenic network in supporting PR activity, whereas *Pax2* is critical for establishing proper retinal structure, and *Oli* is necessary for sustaining neurotransmission and PR activity. Other cell-selective knockdowns suggest that *repo*-positive optic lobe glia, but not CCs, promote histaminergic neurotransmitter recycling (Fig 3C). Notably, while most mature fly glia express Repo, a subset of glia/support cells in peripheral sense organs (sheath, or thecogen, cells) are Repo-negative [77–79,124], yet express both Pros and Pax2 [45,51–53,125–129], much like mature CCs. Moreover, although previous studies on *Drosophila Oli* suggest that this factor—known to participate in gliogenesis in *C*. *elegans* and mice [87,88,94]—was not involved in fly gliogenesis, these conclusions were based on the restricted study of Repo-positive glia [86]. Our finding that *Oli* is an important transiently expressed transcription factor in CCs raises the possibility that this gene could be more generally important in insect glia than previously recognized. Further, given that the *repo* gene has been lost in the vertebrate lineage [130], while *pros*, *Pax2* and *Oli* play deeply conserved glial functions [40,43,47,48,50,51,89,94,131], further examination of roles and the interplay among this ensemble of factors in fly CCs offers a system to uncover both lineage-specific and more deeply conserved “glial character networks” required for promoting neuronal support cell functions. Indeed, interesting context-dependent cross-talk has already been shown for *Prox1* and *Olig* factors during vertebrate neurogenesis [132], and ocular morphometric phenotypes are observed in patients with mutations in *Pax2* [46,133].

### Similarities between insect cone cells and vertebrate glia

In addition to defining a general glial-nature for CCs, the data presented here and elsewhere reveal a striking number of commonalities between insect CCs and vertebrate Müller glia at the developmental, structural, molecular, and functional levels. Many of the support functions identified here in CCs are common to other glial cell types across the nervous system. Nevertheless, both CCs and Müller glia represent specialized macroglial cells that specifically serve retinal neurons. Thus, highlighting these key common features provides a useful framework to further understand shared features with other glial subpopulations.

Developmentally, both CCs and Müller glia are intrinsic to the retina, and adopt their non-neuronal fate from a pool of equipotential neurogenic precursor cells via Notch signaling [108,134,135]. *dPax2* and *pros* are essential for these Notch-dependent decisions in CCs [31], resembling their gliogenic roles in other parts of the fly and vertebrate nervous systems [40,50,51,57,58,136]. Interestingly, orthologs for both factors have also been reported to be expressed in Müller glia [131,137–139], raising the possibility that, like in *Drosophila* CCs, mouse orthologs *Prox1* and *Pax2* may control overlapping but distinct roles in determining Müller glia fate and function in the vertebrate retina.

Morphologically, CCs and Müller glia radially span the retina, in direct contact with all retinal neurons and connecting an apical vitreous-like substance with a basal blood-retina barrier. This is similar to other radial glial subtypes such as Bergmann glia and tanycytes. Additionally, Müller glia [7,140,141] and CCs both have the capacity to serve as intraretinal light guides. With this feature in mind, it is notable that small heat shock/ɑ-crystallin-related proteins are highly enriched in both CCs and Müller glia [142–144] (S3–S5 Tables). This feature is also true for other glia, including oligodendrocytes, and has mainly been attributed to their role as chaperone proteins that provide neuroprotective support [145–147]. This property warrants investigation in *Drosophila* CCs, especially since one small heat shock protein (*hsp23*) is known to be specifically upregulated in CCs under stress conditions [148]. However, it is also attractive to speculate that crystallins in Müller glia and CCs have been co-opted to help mediate light guidance, as has been proposed for the evolution of lenses across the animal kingdom [22, 149].

And finally, at the molecular and functional levels, we find that CCs share considerable gene and physiological overlap with a number of glial types. These include factors commonly used to define Muller glia and are critical for their function [8,150]. In particular, we describe the requirement of conserved gliotypic effector genes such as the Na/K-ATPase, Kir channels, EAAT1, Glut1 and Ldh in CCs for maintaining PR neuronal activity.

It is possible that these detailed similarities between Müller glia and CCs independently evolved in response to common functional requirements of photoreceptors that are inherent to complex eye types. Nevertheless, comparative studies suggest that the earliest eyes in the bilaterian ancestors were minimally comprised of two cells: a photosensitive neuron associated with an accessory pigment cell [22,151–154]. This model is largely based on the requirement of light insulation for directional information, but this two-cell eye type also raises the possibility that essential “neuron-glia” neuroprotective support could have existed in simpler and/or ancestral visual systems [22]. The overlapping functions of CCs, Muller glia, and pigmented epithelial cells lays the groundwork to identify and test the possibility that diverse glial modules existed in sensory systems prior to the separation of vertebrates and invertebrates.

Regardless of the evolutionary origin of CCs, their genetic and functional overlap with vertebrate and invertebrate glial subtypes provides a molecular framework to analyze and identify conserved elements of glial regulatory networks central for establishing and maintaining a healthy sensory system. Moreover, the described capacity to track the progression of neurodegeneration in vivo, using non-invasive, glia- and neuron-specific tools expands the use of the fly retina as an effective system to define deeply conserved mechanisms of neuron-glia biology.

## Materials and methods

### Generation of fly lines

A 275 bp *prospero* eye enhancer, which we called *pros*^*PSG*^, was PCR-amplified from genomic DNA extracted from y*w*^*67*^ flies with the following primers: CCGGAATTCATCTGTGACGAAGACACTCGTTT and CGCGGATCCTCGATTGCCAGGAAGTGC, using previous mapping studies *[155]* as a guide. To generate the *pros*^*PSG*^-GAL4 driver, the *pros*^*PSG*^ fragment was cloned as an EcoRI/BamHI fragment (sites underlined in primers) into hs43GAL4-pCHAB [156]. To create *pros*^*PSG*^-GFP, the *pros*^*PSG*^ enhancer and *hs43* TATA box was PCR-amplified from *pros*^*PSG*^-GAL4 using the primers: CGAGAATTCGGTACCCGCCCGGGATCAGATC and TCGAAGATCTCTGCAGATTGTTTAGCTTGTTCAGCTGC and cloned as an EcoRI/BglII fragment into pStingerAttB-GFP (gift from B. Gebelein, CCHMC) at the EcoRI/BamHI sites (underlined). Transgenic flies were generated using standard procedures (Rainbow Transgenics) in *yw*^*67*^ flies for the GAL4 line or the attB insertion site 51C (containing M{3XP3-RFP.attP’}) for the GFP line.

### Drosophila genetics

The following alleles were used available through the Bloomington Stock Center (BSC) and the Vienna Drosophila Resource Center (VDRC): *gcm*^*1(HM05124) & 2 (JF01074)*^, *repo* ^*1(HMS02971) & 2(JF02974)*^, *Oli*^*1(HMJ02216) & 2(JF02001)*^, *ATPα* ^*1(HMS00703) & 2(JF02910)*^, *nrv2*^*1(HMS01637) & 2(JF03081)*^, *nrv3* ^*1(HMS02961) & 2(JF03367)*^, *Irk2*^*1(HMS02379) & 2(JF01838)*^, *Irk3*^! *(KK107031) & 2(JF02262)*^, *Irk1*^*1(HMS02480) & 2(JF01841)*^, *dLdh*^*1(KK102330) & 2(HMS00039)*^, *Glut1* ^*1(HMS02152) & 2(JF03060)*^, *Eaat1*^*1(KK100187) & 2(HMS02659)*^, *Eaat2* ^*1(KK107989) & 2(HMS01998)*^, *Gs2*^*1(HMS02197) & 2(GD9378)*^, *pyd3*^*1(HMS01029) & 2(GD10029)*^, *ninaE* ^*1(JF01438) & 2(JF01439)*^, *sev*^*14*^, *sv*^*spapol*^, UAS-GFP (BSC stock 5130), FRT82B*ubi*-GFPnls,RpS3 (BSC stock 5627), *repo-GAL4* (BSC stock 7415), *repo*^*1*^ (BSC stock 4162) and *otd*^*1*.*6*^*-GAL4* [156]. Other lines used were: *pros*^*PSG*^-GFP (generated here), *pros*^*PSG*^-GAL4 (generated here), *dPax2*^*RNAi*^ [31], *pros*^*RNAi*^ [31], FRT82B-*pros*^*17*.15^ [157], the retina-specific flippase *ey*^*3*.*5*^*-flp* [158], and the fate-mapping UAS-H2B:YFP reporter [159]. For CC knockdown experiments, flies with the genotype *yw*^67^/*yv*^*1*^; *pros*^*PSG*^-GAL4/+; UAS-RNAi/UAS-GFP flies were analyzed. Other genotypes included *yw*^*67*^; *otd*^*1*.*6*^-GAL4/+; UAS-RNAi/+ (for PR knockdowns), *yw*^*67*^; +/+; *repo*GAL4/UAS-RNAi (for *repo* knockdowns), *sev*^*14*^/+ (control) vs *sev*^*14*^/*sev*^*14*^ (experimental), *repo*^*1*^/+ (control) vs *repo*^*1*^/*repo*^*1*^ (experimental), *yw*^*67*^; *pros*^*PSG*^-GAL4/+; UAS-GFP/+ (controls for *pros* and *dPax2* RNAi knockdowns), *yw*^*67*^; *otd*^*1*.*6*^-GAL4/+; UAS-GFP/+ (controls for *ninaE* RNAi) and *yv*^*1*^; +/+; UAS-RNAi (controls for all remaining RNAi lines). To analyze eyes almost entirely mutant for *pros*, eye-specific Minute clones using the null *pros* allele, *pros*^*17*^, were generated as previously described [31]. Flies were raised on standard cornmeal-molasses food at 25°C in ambient light conditions unless otherwise noted. For *repo>repo*^*RNAi*^ knockdown experiments, animals were either raised at 25°C from time of egg laying (for *repo*^*null*^-like phenotypes), or were raised at 18°C until late 3rd instar stages and then shifted to 25°C (for *repo*^*1*^-like phenotypes).

### Photoreceptor degeneration, histology and microscopy

For light-induced degeneration, 25 flies per experimental group were raised 12 inches from a 25-watt fluorescent light bulb for 7 days. In three separate experiments, daily analysis was conducted to quantity the number of flies with an intact deep pseudopupil [73,160,161] as an indicator of intact photoreceptor rhabdomeres. Statistical comparisons were performed using 1-way ANOVA. At the end of the 7 day experimental period, eyes were dissected, fixed and prepared for semi-thin plastic sections or whole mount phalloidin staining. For plastic and thin sections, adult eyes were dissected in PBS, fixed in 4% formaldehyde in PBS at RT for 30 min and post-fixed in 2% osmium tetroxide in PBS on ice for 60 min. Tissue was serially dehydrated in EtOH and infused with LR-white resin (EMS) overnight. The resin was polymerized in gelatin capsules at 65°C overnight. For semi-thin sections, 1μM sections were placed onto glass slides, stained with toluidine blue (CCHMC Pathology Core) and imaged on a Zeiss Axioplan2. Thin EM sections were mounted on 200 mesh copper grids and stained with 2% uranyl acetate and lead citrate [162,163] (CCHMC Pathology Core) and imaged on a Hitachi H7650 TEM. For immunofluorescence, eyes were dissected in PBS, and either fixed in 4% formaldehyde at RT for 15 min or in -20°C methanol overnight followed by immunostaining procedures previously described [31]. Antibody concentrations used were: GFP (rabbit, 1:500, Invitrogen), GFP (goat, 1:500, Abcam), Elav (rat,1:200, DSHB), Fas3 (mouse,1:50, DSHB), ATPα (mouse, 1:50, DSHB), Nrv1 (guinea pig, 1:150, Paul et al, 1998), Nrv2 (rabbit, 1:150, Sun et al, 1998), Nrv3 (rabbit, Baumann et al. 2010), Repo (mouse, 1:20, DSHB), 22C10 (mouse, 1:50, DSHB), β-alanine (rabbit, 1:1000, Abcam). Donkey secondary antibodies were conjugated to AlexaFluor 488, 555 or 647 (Invitrogen). Actin-rich rhabdomeres were detected using AlexaFluor 555-conjugated phalloidin (1:50, Invitrogen). Samples were imaged on a Nikon A1R multiphoton confocal, and image processing was performed using NIS-elements (Nikon), Imaris (bitplane) and Photoshop CC (Adobe).

### Electroretinograms

Unless otherwise stated, 1-day old flies were immobilized with CO_2_, individually mounted onto plastic coverslips with dental wax, and dark adapted for 30 minutes. The electrophysiological setup included a faraday cage, a 1600 AM-Systems amplifier (Sequim, WA, USA), and an iWorks AD board 118 with LabScribe2 software (iWorks Systems, Dover, NH, USA). The recording electrode (a PBS-soaked cotton wick connected to a silver wire) was positioned on the surface of the eye, and the grounding electrode (a PBS-soaked glass electrode connected to a silver wire) was placed between the third and fourth abdominal segments. Signals were sampled at 10,000 Hz. To elicit responses from all PRs except the UV-sensitive Pros-positive R7s, 5 pulses (5secs on, 15sec off) of blue-green light (490nm LED, LED supplies part #L4-0-T5TH15-1) were delivered via a fiber optic cable positioned immediately adjacent to the eye, delivering a light intensity of 3.55 x 10^14^ photons/cm^2^/sec. Data was collected from at least five flies per genotype from one RNAi line and three flies from a second RNAi line, with controls (GAL4 or RNAi lines) being recorded on the same day to avoid day-to-day variation. All genotypes were also initially tested using a white LED light delivering a light intensity of 2.77 x 10^15^ photons/cm^2^/sec, with similar results observed. To confirm that any reduction in photoreceptor response-strength was not simply due to a shift in relative light sensitivity, VlogI curves were generated with 150 msec pulses and 30s recoveries at different light intensities, with all reported recordings performed within the linear range of these curves (S5B Fig). All data was analyzed with a custom Matlab program with the following parameters. First, data was first smoothed [filter{ones(1,windowsize)/windowsize,1 data}] with a window size of 10. The PR response amplitude was calculated as the absolute voltage difference between the average of 100 baseline values (immediately prior to stimulation) and the amplitude of the sustained negative response, measured as the average of 100 points immediately prior to stimulus termination. All test PR responses reported in Figs 3B, 3C and 5 were normalized to day and genotype-matched controls (GAL4 or RNAi lines, described above). Raw values are plotted in S4 Fig. The “on” transient amplitude was calculated as the absolute voltage difference between the baseline and the maximum voltage reached during stimulation. We confirmed that these transients were linear with respect to PR activity at the light levels used in these experiments [164,165]. Therefore, to exclude the possibility that reduced transients did not merely reflect decreased PR receptor responses, relative transient size was calculated based on the ratio of the “on” transient strength to the maximal PR response. To assess changes in “on” transients in response to repeated stimuli, the ratio of the first and last transients was calculated. Absolute voltage values were normalized as percentages relative to day and genotype (RNAi or GAL4 alone)-matched controls (Fig 3C). All captured data followed a normal distribution by Kruskal-Wallis tests. Stated significance was determined using multiple t-tests between each sample and its day/genotype-matched control. p-values were corrected using Benjamini and Hochberg’s false discovery rate (Microsoft Excel and Prism v6).

### FACS, RNA isolation and RNAseq

For cell sorting experiments, eye tissue (from which antennae, brain and lamina tissue were carefully removed) from 45–75 animals was dissected from larva (wandering late third instars), pupae (25% after puparium formation [25 hr at 25°C]) and adults (1–2 days post-eclosion) in ice-cold PBS during a period no longer than 2 hours. Tissue was dissociated by placing whole eyes in 0.5% trypsin for 15 min at room temperature. Single cell suspensions were made by pipetting dissociated tissue in PBS/1% FBS (Gibco)/1mM EDTA with a P200 tip coated with 1% BSA. For larval (L) and pupal (P) cone cells (CCs), GFP-positive cells were sorted from R7-less *sev*^-^; *pros*^*PSG*^-GFP; TM2/TM6B animals. For adult (A) CCs, dissected whole-mounted retinas from *yw*^*67*^;sp/CyO;TM2/TM6B flies were dissociated and stained 10 min with pre-conjugated Fas3-Alexa555 (1:50) to mark CCs and 22C10-Alexa647 (1:50) to mark the photoreceptors and interommatidial bristle (Fig 1G). Control sorts from unstained samples were performed using *sev*^-^; sp/CyO; TM2/TM6B animals for larval and pupal stages and *yw*^*67*^;sp/CyO;TM2/TM6B flies for adults. Fas3 (7G10) and 22C10 monoclonal antibodies were developed by Corey Goodman [166] and Seymour Benzer [167], and obtained from the NIH/NICHD-created Developmental Studies Hybridoma Bank maintained at The University of Iowa, Department of Biology, Iowa City, IA 52242. Antibody conjugations were performed using APEX labelling (Invitrogen) according to manufacturer’s suggestions. Photoreceptor RNAseq data was obtained from RFP-positive/GFP-negative gated cells from adult *pros*^*PSG*^-GFP retinas (brain and lamina removed), making use of the attB docking site carrying the 3xP3-RFP transgene that is expressed in adult PRs [168,169].

Cells were sorted directly into lysis buffer and RNA was immediately extracted using the RNeasy Micro kit (Qiagen, cat. 74004) and kept frozen at -80°C. RNA concentration and quality was assessed on an Agilent Bioanalyzer (CCHMC Microarray Core). RNA amplification and cDNA synthesis was performed using the Ovation RNA Amplification System V2 (NuGEN, cat. 7102–08) using manufacturer’s suggestions. Nextera library preparations was performed by the CCHMC Microarray Core and sequenced by the CCHMC DNA Sequencing Core using an Illumina HiSeq2500, with 5–30 million reads dedicated to each sample. Sequencing files have been deposited in NCBI's Gene Expression Omnibus and are accessible through GEO Series accession number GSE93782.

### Bioinformatics

NGS-pipeline data (provided by the CCHMC Bioinformatics Core) of ~15 million reads per sample were mapped to the *Drosophila* genome (Dm3) with >78% of the reads uniquely mapped. A pooled meta-analysis (F-test, p-value 0.05) was performed on genes within the 10-100th percentile of the RPKM (S1 Table) using Avadis NGSv1.6 software. The trimmed mean of M-values (TMM) normalization method [170] was used to remove RNA compositional biases between samples with different experimental and sequencing conditions and to improve the compatibility of cross-sample analysis. All samples were normalized to whole animal transcriptome data, and pooled meta-analysis and read density thresholding was used to remove genes that fell below statistically significant levels of expression (<2 NRPKM, p<0.05). This allowed analysis of ~6,500 genes expressed at each stage of CC development (S1 Table). Validation of the integrity of cell specificity and sequencing was confirmed by comparing the adult CC transcriptome with a second, independently prepared *Drosophila* adult CC library. Strong agreement between the two sequencing data sets was observed (R^2^ = 0.84) (S2A’ Fig). For intra- and inter-species analysis, enhanced gene sets for CCs, PRs, CNS (from modEncode sample #5312), and digestive system (DS, from modEncode sample #3445) were calculated based on ranked relative differential (square-root) expression of cell-type specific transcripts as compared to adult whole fly transcriptome data (NCBI GSM 694258–61: average of 2 males and 2 female) (S2 Table) [171,172]. For comparison of enhanced sets across species, sets of 1000 genes that most clearly differentiated CCs from PRs (and vice versa) (CC>PR and PR>CC, respectively) were calculated based on a ranked list of divergence using the differential of square root TMM expression values between these two cell types (S2 Table). Fly-to-mouse gene conversions were performed using the DRSC Integrative Ortholog Prediction tool (version 5.1.1) [173], using the criteria: only genes with score size >2 unless only match score is 1 or 2 and the ortholog is represented the best score in fly-to-mouse or mouse-to-fly direction (S4 Table). Enriched gene sets from mouse neural cell types were based on previous Dropseq analysis of retinal cells [106] and microarray analysis of isolated forebrain astrocytes, oligodendrocytes and cortical neurons [107]. From the microarray studies, astrocytes, oligodendrocytes and cortical neuron-enriched genes were defined as being statistically enriched (FDR<1%) by at least 1.5 fold in each cell type [107]. Gene identifiers from all sets were converted to Entrez IDs for cross experiment analysis using the 5/1/2016 update of NCBI Gene IDs of WebGestalt [174] (S5 Table). Overlap between gene sets was analyzed using Venny (http://bioinfogp.cnb.csic.es/tools/venny/) [175], with statistical significance of overlap calculated based on normal approximation of hypergeometric probability [176].

## Supporting information

S1 Fig (Related to Fig 1). Expression of prospero, dPax2 and the *pros*^*PSG*^ enhancer in adult cone cells. **(A)** Prospero (Pros, magenta) (A’) and dPax2 (green) (A”) antibody staining in whole mounted adult retinas shows their co-expression in mature CCs. (**B)** Cryosections from adult heads in which cells expressing the *pros*^*PSG*^-GAL4 driver were fate-mapped with a UAS-H2B:YFP reporter (*pros*^*PSG*^*-GAL4>UAS-H2B*:*YFP)*. YFP [green], Pros [blue] and Elav [red] shows highly restricted and strong staining in R7 photoreceptors (white arrow) and CCs (yellow arrowhead), with little to no staining in the underlying optic lobe (retina-brain barrier marked with dotted white line). (**C**) Adult head cryosections from *pros*^*PSG*^-nGFP flies (green) similarly shows restricted expression to the CCs, weak R7 expression, and no detectable expression in cells the underlying brain [lamina (L) or optic lobe (O)] (nuclei marked with Hoechst, blue).
(TIF)

S2 Fig (Related to Fig 2). *prospero* and *dPax2* in CCs affects photoreceptor morphology and functional integrity. **(A-C)** Phalloidin-stained sagittal cryosections from adult control (*pros*^*PSG*^-GAL4>UAS-*nGFP*, A), *pros*^*RNAi*^ (*pros*^*PSG*^-GAL4>UAS-*pros*^*RNAi*^, B) and *Pax2*^*RNAi*^ (*pros*^*PSG*^-GAL4>UAS-*dPax2*^*RNAi*^, C) eyes. Control and *pros*^*RNAi*^ eyes have outer photoreceptor rhabdomeres that extend through the full depth of the retina, and an actin-rich fenestrated membrane (dashed line) separating the retina from the brain (A,B). In *dPax2*^*RNAi*^ eyes, retinal organization is severely disrupted, with incomplete elongation of rhabdomeres and some PRs misplaced into the brain (C), having fallen through the retinal floor (arrows). **D)** Normalized PR activity from light- or dark-adapted flies, measured by ERGs. Similar reductions in activity are observed in dark-adapted or *pros*^*17*^ mutants flies (*pros>pros*^*RNAi*^ or eyFLP; FRT82-*pros*^*17*^/FRT82-Minute clones). An even further reduction in PR activity is observed in *pros*^*RNAi*^ flies prior dark adaptation (light-adapted). *p<0.001 **E-J)** PR rhabdomere structure, visualized by toluidine blue semi-thin sections (E-G) or phalloidin staining of adult whole mount eyes (H-J) shows that control flies raised in constant light (E,H) or *pros*^*RNAi*^ flies raised in total darkness (F,I) are similarly intact, whereas *pros*^*RNAi*^ flies raised in constant light for 7 days (G,J) show degeneration.
(TIF)

S3 Fig (Related to Figs 2, 3 and 5): Photoreceptor activity and “on” transient analysis of cell-selective RNAi knockdowns and mutants. Calculations from ERG recordings for the sustained negative response (PR activity) (**A, B**), “on” transient size average (n = 5 flies, 5x5sec light pulses), normalized to PR activity (**C**), or activity-dependent changes in “on” transients (taken from last light pulse—first light pulse(**D**). Day-matched controls (black) were included for each experimental condition (labeled, grey). PSG = *pros*^*PSG*^*-GAL4*; O2 = otd^1.6^-GAL4, line #2; repo = repo-GAL4. *p<0.001.
(TIF)

S4 Fig (Related to Fig 3): Transcriptome and *in vivo* expression analysis of CC-expressing genes. **(A)** Representative FACS analysis of adult CCs and PRs (left). PRs were labeled with m22C10-conjugated to AlexaFluor555, and CCs were labeled with anti-Fas3 conjugated to AlexaFluor488. Unlabeled retinal cells from *yw67; Sp/Cyo; TM2/TM6B* flies served as a negative control (right). **(A’)** Comparison of overall transcript expression values between cell types (larval, pupal, and adult CCs, as well as adult PRs), based on TMM normalized counts (log_2_) of 14182 genes. Adult x adult CC plot compares the transcript counts for the adult CC dataset used in the manuscript with an external cone cell RNA-seq data set generated using the same approach but at later date. Parallel alignment strategies were employed, with alignment to dm6 (16823 transcripts). For these separately sequenced sets, transcript counts were normalized to 1M based on total aligned reads. R^2^ values for all comparative plots are based on log-scaled values to minimize effect of few transcripts with very high read counts. **(B)** TMM-normalized log_2_ mRNA expression levels from late larval, early pupal, and adult CCs as well as adult PRs. Common housekeeping genes (*GAPDH1*, *GAPDH2*, *Rp13A*, *RpL32*, *and RpLP0*) are approximately equally represented in all 4 cell populations, whereas genes with known PR-restricted expression (*ocelliless [otd]*, *ninaE [Rh1]*, *Rh3*, *Rh4*, *Rh5*, *Rh6*, *Arrestin1 (Arr1)*, and *chaoptin (chp)* are highly enriched in the PR transcriptome with little to no expression in CC transcriptomes. **(C,D)** Expression of *gcm*-LacZ (green) in Cut-positive cone cells (magenta) from larval (late 3^rd^ instar) and pupal (8 hr after puparium formation [APF]) eye tissue. Weak expression is detected in a subset of CCs at late stages of larval development (dotted circles, C, C‘) and is obvious in all 4 CCs by early pupation (dotted circles, D, D‘). A positive control for *gcm*-LacZ expression the optic stalk is indicated by an arrow (C, C‘). No detectable *gcm*-LacZ was in CCs after this stage of development. **(E,F)** Immunostaining of Repo (green), and counterstained with Pax2 (magenta) reveals weak expression in control CCs by 25 hr APF (circles, E, E‘), which is not detected in *repo*^*RNAi*^ knockdowns (F,F‘). Expression in the interommatidial bristle lineage (arrows) is detected in both conditions, providing further support for the specificity of the knockdown approach.
(TIF)

S5 Fig (Related to Figs 3 and 5): Electrophysiological analysis of cell-specific knockdowns, mutants, and controls. **A**) ERG plots (overlay of five consecutive pulses) from individual, representative flies with noted genotypes. **B**) VlogI curves were produced in each CC knockdown to establish the dynamic range of photoreceptors. Data was fit to the Naka-Rushton (NR) function V/Vmax *In/(In+Kn) [177]. I is the stimulus intensity, V corresponds to the measured response amplitude, and Vmax, K and n are constants (corresponding to the maximum response amplitude, the stimulus intensity that elicits half of the maximum response and the slope of the function, respectively). Light intensities ranged from 2.86 x 101^1^ to 1.7 x10^15^ photons/cm^2^/sec. Dashed lines indicate light intensity used for this study (3.55 x 10^14^ photons/cm^2^/sec).
(TIF)

S6 Fig (Related to Fig 5): Immunohistochemical analysis of cell-specific knockdowns. **(A-B)** Immunostaining of whole-mount adult eyes from control (C, *pros>nGFP*) or *nrv3* CC knockdowns (*pros>nrv3*^*RNAi-1*^) shows CC-restricted knockdown of the Nrv3 subunit of the Na/K pump (A‘ vs B‘, magenta), whereas the Nrv2 subunit (A,B, green) and α-subunit (ATPα, A“, B”blue) maintain their expression in both genetic backgrounds. **(C-E)** β-alanine immunostaining (green) is reduced in flies in which *pyd3* is knocked down in CCs (*pros>pyd3*^*RNAi-1*^*)*, D), but is still present in the pseudocone from control (*pros>nGFP*, C) flies or in flies where the *pyd3*^*RNAi-1*^ transgene is driven in photoreceptors (*otd*>*pyd3*^*RNAi-1*^, E). Fas3 (magenta) is used to mark the CC layer.
(TIF)

S1 Table (Related to Fig 3). TMM normalized gene expression levels from Drosophila cell types. Transcriptomes from larval, pupal and adult cone cells, adult photoreceptors (described in the current study), as well as publicly-available transcriptomes from the Drosophila central nervous system and digestive system [172,178] were TMM-normalized to whole animal gene expression levels and represented as log2 values.
(XLSX)

S2 Table (Related to Fig 3). Enriched *Drosophila* gene sets used for intra- and inter-species glial gene analysis. Genes from S1 Table sorted by relative gene expression levels from different cell populations. The top 1000 genes for the analysis in the current study are highlighted.
(XLSX)

S3 Table (Related to Fig 3): *Drosophila* glial gene sets used for Drosophila intra-species analysis. Gene lists from 109 genetically confirmed glia-associated factors [179] and 2309 genes showing expression change in both *gcm* loss- and gain-of-function animals (derived from [180]).
(XLSX)

S4 Table (Related to Fig 4): Gene sets used for analysis between Drosophila and murine cell types. Fly-to-mouse DIOPT conversions of the top 1000 CC- or PR-enriched datasets (CC>PR and PR>CC from S2 Table) used for cross-species analysis.
(XLSX)

S5 Table (Related to Fig 4): Gene sets used for analysis between Drosophila and murine cell types. Gene sets from murine retinal and forebrain neural cell types [106,181] used for overlap analysis with genes enriched in Drosophila cone cells and photoreceptors. Genes highlighted in green represent genes whose fly orthologs are enriched in Drosophila CCs, while those highlighted in blue represent those with fly orthologs enriched in PRs.
(XLSX)
