# Supplementary figures and images for: Multifunctional glial support by Semper cells in the Drosophila retina
Source: PLoS Genet. 2017 May 31;13(5):e1006782. doi: 10.1371/journal.pgen.1006782 (PMC5470715; doi:10.1371/journal.pgen.1006782)

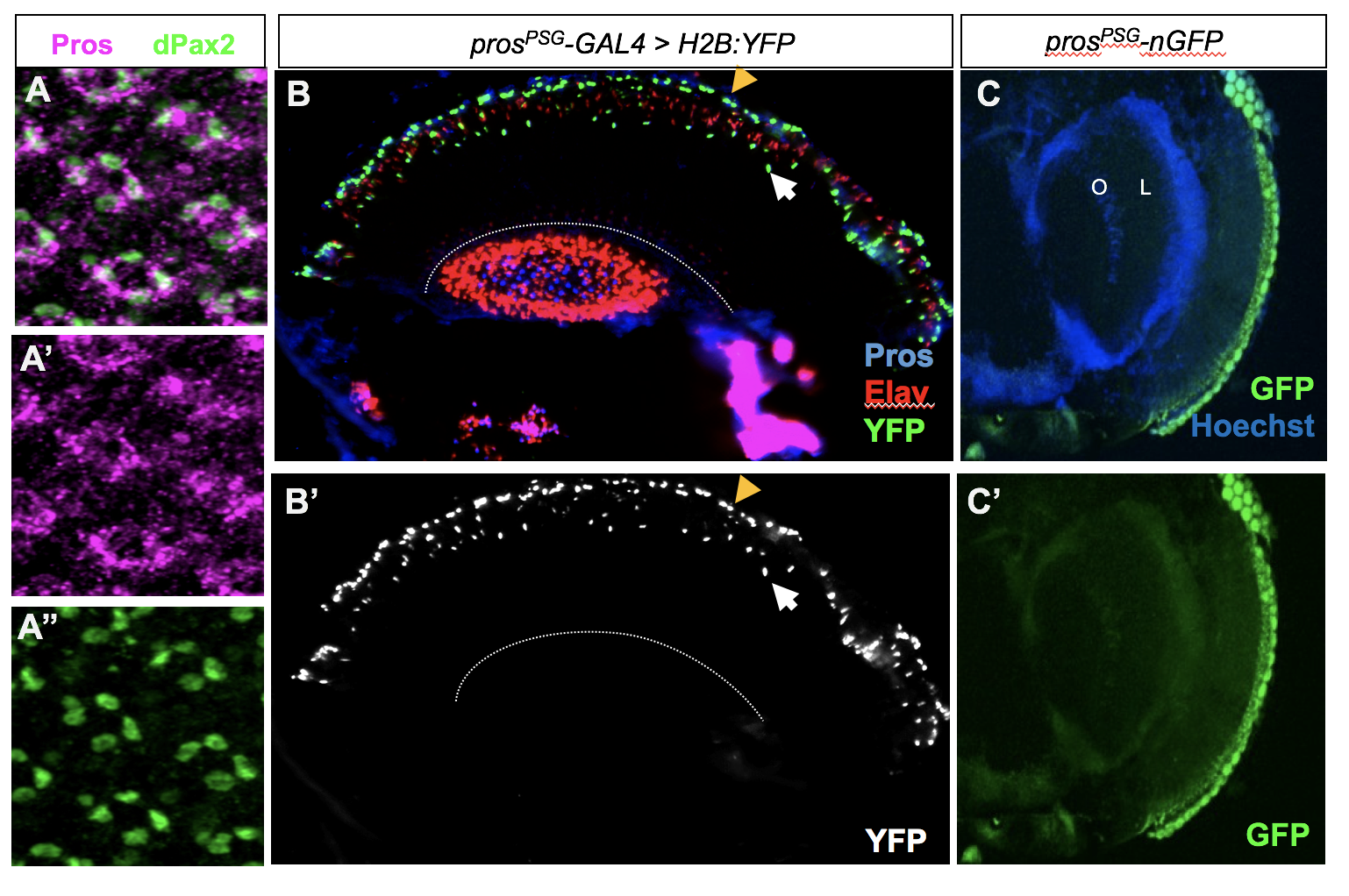

Supplement: S1 Fig — (A) Prospero (Pros, magenta) (A’) and dPax2 (green) (A”) antibody staining in whole mounted adult retinas shows their co-expression in mature CCs. (B) Cryosections from adult heads in which cells expressing the prosPSG-GAL4 driver were fate-mapped with a UAS-H2B:YFP reporter (prosPSG-GAL4>UAS-H2B:YFP). YFP [green], Pros [blue] and Elav [red] shows highly restricted and strong staining in R7 photoreceptors (white arrow) and CCs (yellow arrowhead), with little to no staining in the underlying optic lobe (retina-brain barrier marked with dotted white line). (C) Adult head cryosections from prosPSG-nGFP flies (green) similarly shows restricted expression to the CCs, weak R7 expression, and no detectable expression in cells the underlying brain [lamina (L) or optic lobe (O)] (nuclei marked with Hoechst, blue). (TIF) [file pgen.1006782.s001.tif]

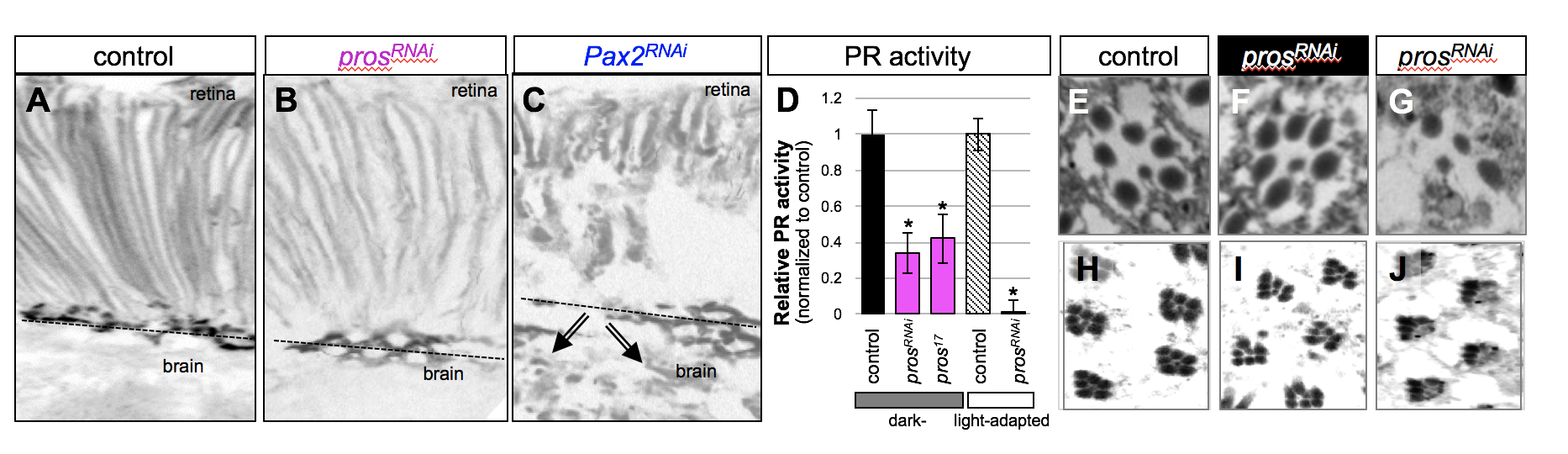

Supplement: S2 Fig — (A-C) Phalloidin-stained sagittal cryosections from adult control (prosPSG-GAL4>UAS-nGFP, A), prosRNAi (prosPSG-GAL4>UAS-prosRNAi, B) and Pax2RNAi (prosPSG-GAL4>UAS-dPax2RNAi, C) eyes. Control and prosRNAi eyes have outer photoreceptor rhabdomeres that extend through the full depth of the retina, and an actin-rich fenestrated membrane (dashed line) separating the retina from the brain (A,B). In dPax2RNAi eyes, retinal organization is severely disrupted, with incomplete elongation of rhabdomeres and some PRs misplaced into the brain (C), having fallen through the retinal floor (arrows). D) Normalized PR activity from light- or dark-adapted flies, measured by ERGs. Similar reductions in activity are observed in dark-adapted or pros17 mutants flies (pros>prosRNAi or eyFLP; FRT82-pros17/FRT82-Minute clones). An even further reduction in PR activity is observed in prosRNAi flies prior dark adaptation (light-adapted). *p<0.001 E-J) PR rhabdomere structure, visualized by toluidine blue semi-thin sections (E-G) or phalloidin staining of adult whole mount eyes (H-J) shows that control flies raised in constant light (E,H) or prosRNAi flies raised in total darkness (F,I) are similarly intact, whereas prosRNAi flies raised in constant light for 7 days (G,J) show degeneration. (TIF) [file pgen.1006782.s002.tif]

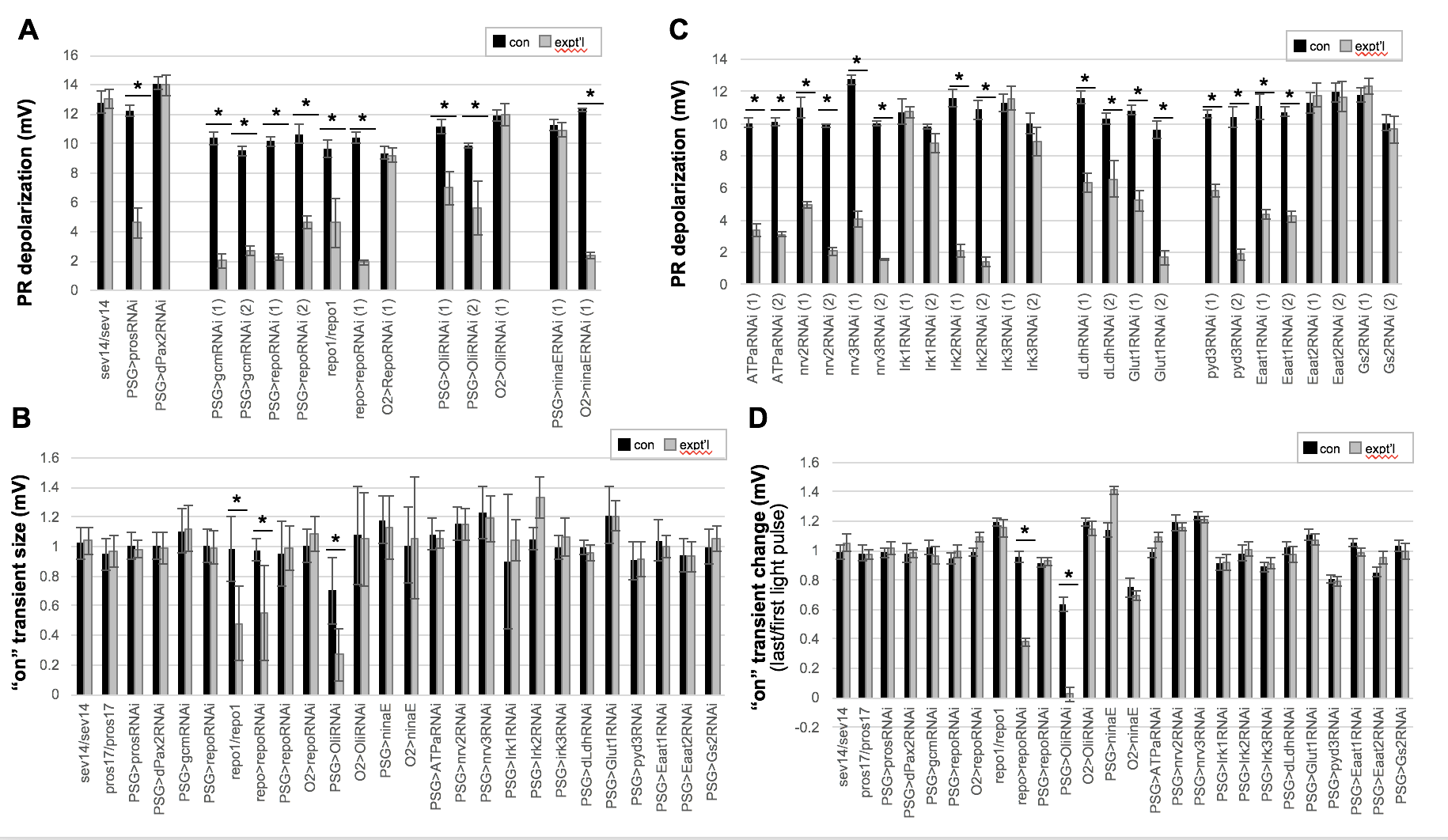

Supplement: S3 Fig — Calculations from ERG recordings for the sustained negative response (PR activity) (A, B), “on” transient size average (n = 5 flies, 5x5sec light pulses), normalized to PR activity (C), or activity-dependent changes in “on” transients (taken from last light pulse—first light pulse(D). Day-matched controls (black) were included for each experimental condition (labeled, grey). PSG = prosPSG-GAL4; O2 = otd1.6-GAL4, line #2; repo = repo-GAL4. *p<0.001. (TIF) [file pgen.1006782.s003.tif]

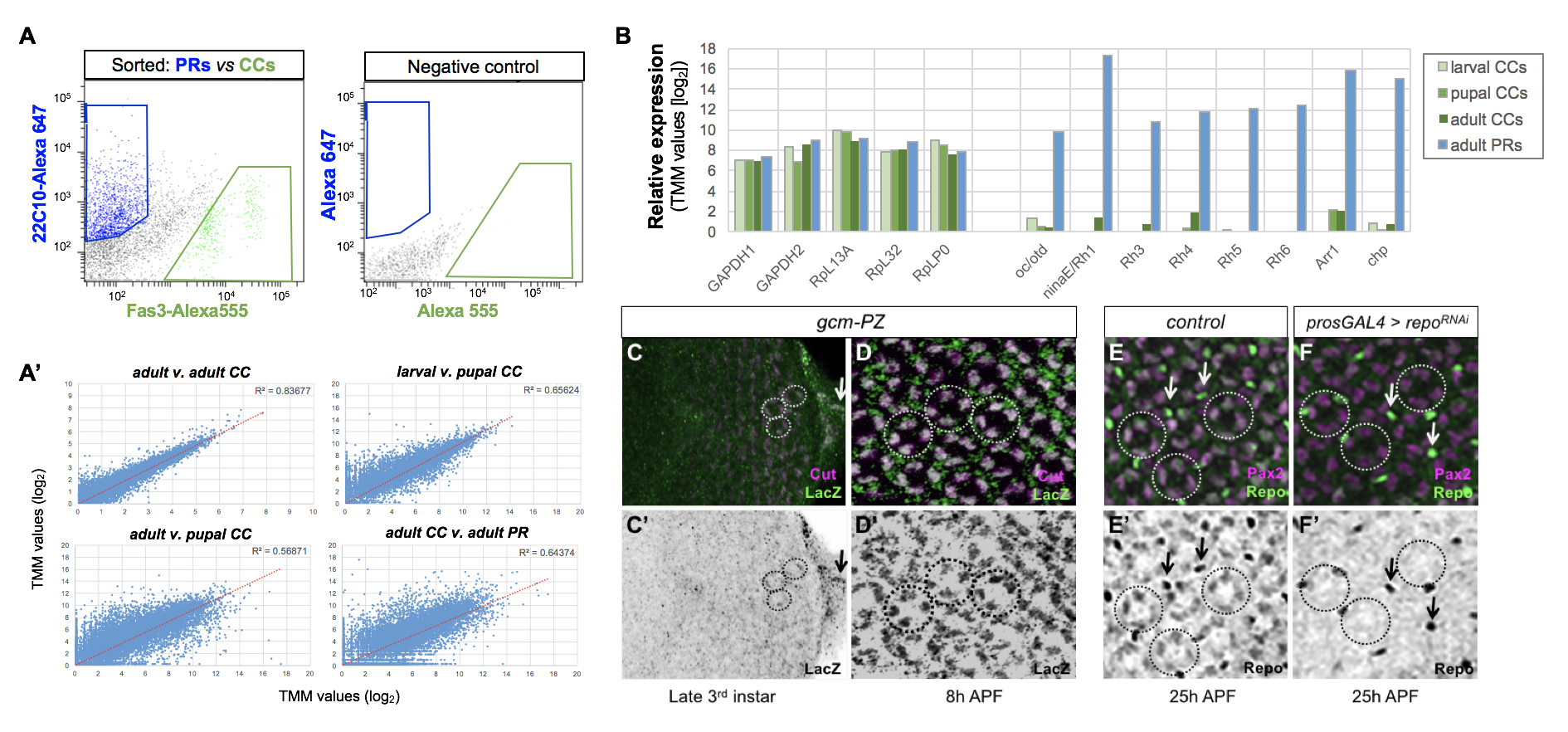

Supplement: S4 Fig — (A) Representative FACS analysis of adult CCs and PRs (left). PRs were labeled with m22C10-conjugated to AlexaFluor555, and CCs were labeled with anti-Fas3 conjugated to AlexaFluor488. Unlabeled retinal cells from yw67; Sp/Cyo; TM2/TM6B flies served as a negative control (right). (A’) Comparison of overall transcript expression values between cell types (larval, pupal, and adult CCs, as well as adult PRs), based on TMM normalized counts (log2) of 14182 genes. Adult x adult CC plot compares the transcript counts for the adult CC dataset used in the manuscript with an external cone cell RNA-seq data set generated using the same approach but at later date. Parallel alignment strategies were employed, with alignment to dm6 (16823 transcripts). For these separately sequenced sets, transcript counts were normalized to 1M based on total aligned reads. R2 values for all comparative plots are based on log-scaled values to minimize effect of few transcripts with very high read counts. (B) TMM-normalized log2 mRNA expression levels from late larval, early pupal, and adult CCs as well as adult PRs. Common housekeeping genes (GAPDH1, GAPDH2, Rp13A, RpL32, and RpLP0) are approximately equally represented in all 4 cell populations, whereas genes with known PR-restricted expression (ocelliless [otd], ninaE [Rh1], Rh3, Rh4, Rh5, Rh6, Arrestin1 (Arr1), and chaoptin (chp) are highly enriched in the PR transcriptome with little to no expression in CC transcriptomes. (C,D) Expression of gcm-LacZ (green) in Cut-positive cone cells (magenta) from larval (late 3rd instar) and pupal (8 hr after puparium formation [APF]) eye tissue. Weak expression is detected in a subset of CCs at late stages of larval development (dotted circles, C, C‘) and is obvious in all 4 CCs by early pupation (dotted circles, D, D‘). A positive control for gcm-LacZ expression the optic stalk is indicated by an arrow (C, C‘). No detectable gcm-LacZ was in CCs after this stage of development. (E,F) Immunostaining of Re [file pgen.1006782.s004.tif]

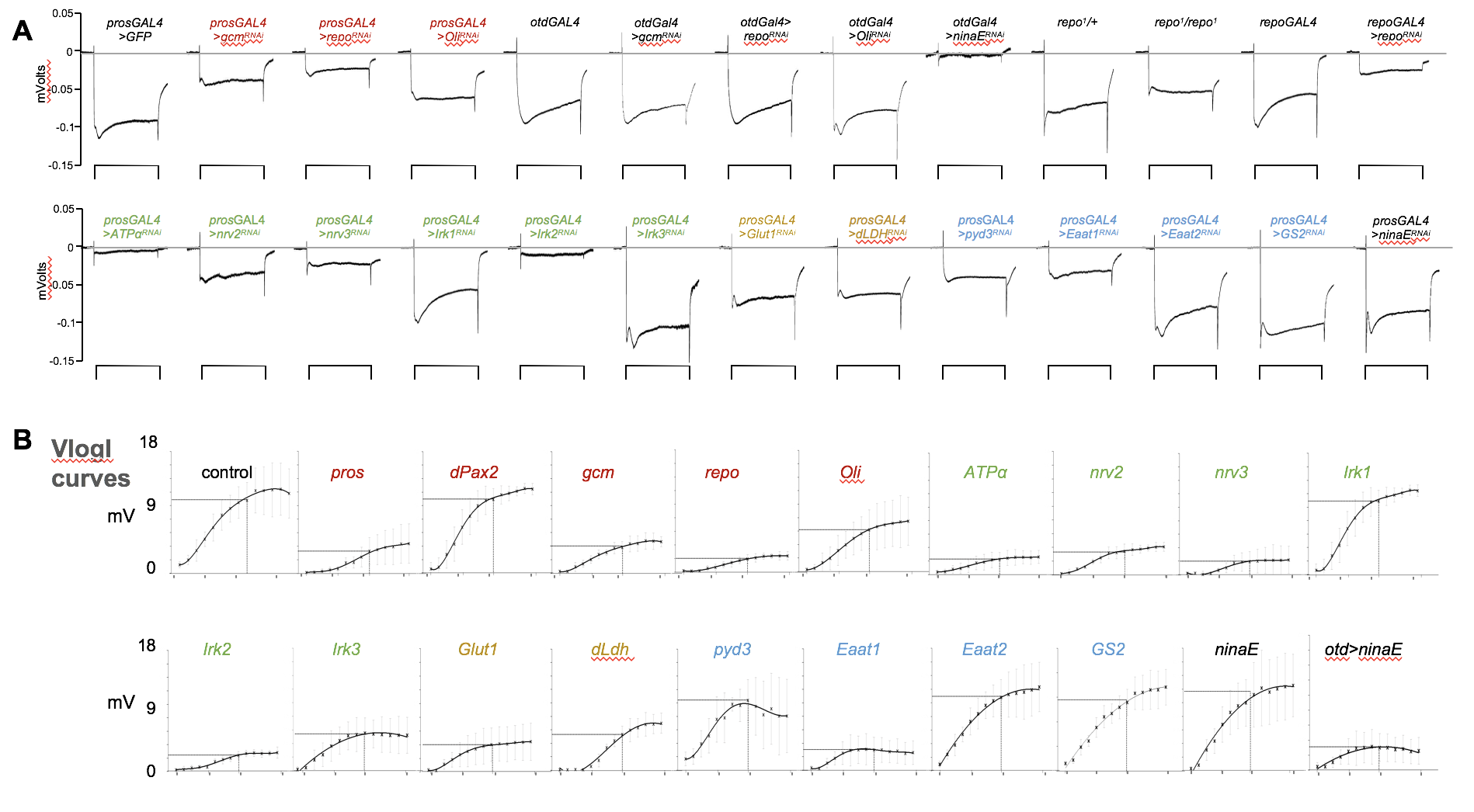

Supplement: S5 Fig — A) ERG plots (overlay of five consecutive pulses) from individual, representative flies with noted genotypes. B) VlogI curves were produced in each CC knockdown to establish the dynamic range of photoreceptors. Data was fit to the Naka-Rushton (NR) function V/Vmax *In/(In+Kn) [177]. I is the stimulus intensity, V corresponds to the measured response amplitude, and Vmax, K and n are constants (corresponding to the maximum response amplitude, the stimulus intensity that elicits half of the maximum response and the slope of the function, respectively). Light intensities ranged from 2.86 x 10^11^ to 1.7 x10^15^ photons/cm2/sec. Dashed lines indicate light intensity used for this study (3.55 x 10^14^ photons/cm2/sec). (TIF) [file pgen.1006782.s005.tif]

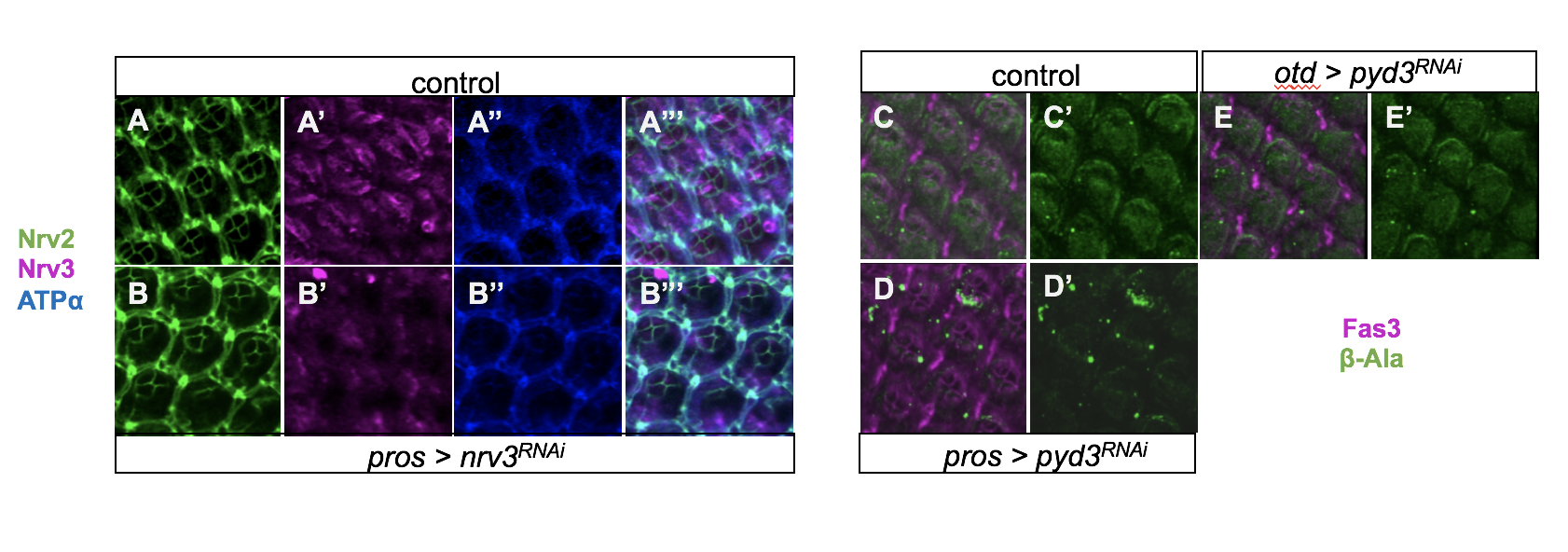

Supplement: S6 Fig — (A-B) Immunostaining of whole-mount adult eyes from control (C, pros>nGFP) or nrv3 CC knockdowns (pros>nrv3RNAi-1) shows CC-restricted knockdown of the Nrv3 subunit of the Na/K pump (A‘ vs B‘, magenta), whereas the Nrv2 subunit (A,B, green) and α-subunit (ATPα, A“, B”blue) maintain their expression in both genetic backgrounds. (C-E) β-alanine immunostaining (green) is reduced in flies in which pyd3 is knocked down in CCs (pros>pyd3RNAi-1), D), but is still present in the pseudocone from control (pros>nGFP, C) flies or in flies where the pyd3RNAi-1 transgene is driven in photoreceptors (otd>pyd3RNAi-1, E). Fas3 (magenta) is used to mark the CC layer. (TIF) [file pgen.1006782.s006.tif]
